# Innate Immune Recognition of *Yersinia pseudotuberculosis* Type III Secretion

**DOI:** 10.1371/journal.ppat.1000686

**Published:** 2009-12-04

**Authors:** Victoria Auerbuch, Douglas T. Golenbock, Ralph R. Isberg

**Affiliations:** 1 Department of Molecular Biology and Microbiology, Tufts University School of Medicine, Boston, Massachusetts, United States of America; 2 Department of Infectious Diseases and Immunology, University of Massachusetts Medical School, Worcester, Massachusetts, United States of America; 3 Howard Hughes Medical Institute, Tufts University School of Medicine, Boston, Massachusetts, United States of America; University of Toronto, Canada

## Abstract

Specialized protein translocation systems are used by many bacterial pathogens to deliver effector proteins into host cells that interfere with normal cellular functions. How the host immune system recognizes and responds to this intrusive event is not understood. To address these questions, we determined the mammalian cellular response to the virulence-associated type III secretion system (T3SS) of the human pathogen *Yersinia pseudotuberculosis*. We found that macrophages devoid of Toll-like receptor (TLR) signaling regulate expression of 266 genes following recognition of the *Y. pseudotuberculosis* T3SS. This analysis revealed two temporally distinct responses that could be separated into activation of NFκB- and type I IFN-regulated genes. Extracellular bacteria were capable of triggering these signaling events, as inhibition of bacterial uptake had no effect on the ensuing innate immune response. The cytosolic peptidoglycan sensors Nod1 and Nod2 and the inflammasome component caspase-1 were not involved in NFκB activation following recognition of the *Y. pseudotuberculosis* T3SS. However, caspase-1 was required for secretion of the inflammatory cytokine IL-1β in response to T3SS-positive *Y. pseudotuberculosis*. In order to characterize the bacterial requirements for induction of this novel TLR-, Nod1/2-, and caspase-1-independent response, we used *Y. pseudotuberculosis* strains lacking specific components of the T3SS. Formation of a functional T3SS pore was required, as bacteria expressing a secretion needle, but lacking the pore-forming proteins YopB or YopD, did not trigger these signaling events. However, nonspecific membrane disruption could not recapitulate the NFκB signaling triggered by *Y. pseudotuberculosis* expressing a functional T3SS pore. Although host cell recognition of the T3SS did not require known translocated substrates, the ensuing response could be modulated by effectors such as YopJ and YopT, as YopT amplified the response, while YopJ dampened it. Collectively, these data suggest that combined recognition of the T3SS pore and YopBD-mediated delivery of immune activating ligands into the host cytosol informs the host cell of pathogenic challenge. This leads to a unique, multifactorial response distinct from the canonical immune response to a bacterium lacking a T3SS.

## Author Summary

Most multicellular organisms have immune sensors that recognize molecules common among microorganisms. Recognition of such molecules informs the host that invading microbes are present, triggering an immune response. Many known innate immune sensors, however, do not appear to distinguish commensals from pathogens. This is in spite of the fact that the host must clear pathogens while simultaneously avoiding a response to benign or beneficial microbes. There are few molecular explanations for how this discrimination occurs in mammalian hosts. To address this problem, we analyzed the response of mammalian cells to the gut pathogen *Yersinia pseudotuberculosis*. We found that *Yersinia* expressing a virulence-associated secretion system caused a transcriptional response in host cells that was very different from the response to a strain with a nonfunctional version of the secretion system. This transcriptional response included several distinct signaling pathways leading to production of mediators of innate immunity, including cytokines such as type I interferon and TNF-α. A large number of pathogens express specialized secretion systems similar to that in *Yersinia*, so these findings provide evidence that there is a mammalian immune response to alterations in host cells that results from pathogen attack, supporting known systems for recognition of common microbial molecules.

## Introduction

The ability to detect pathogens while maintaining beneficial commensal bacteria is important for the health of animal hosts [1]. In order to defend itself against potentially injurious intruders, the host must recognize and respond to previously unencountered microbes. This is achieved in part by recognition of molecules unique to microbes but common among subgroups of bacteria, viruses, or fungi [2],[3]. Detection is accomplished partially by a group of 13 mammalian surface-associated proteins called Toll-like receptors (TLRs) which induce an immune response upon recognition of microbial molecules such as lipopolysaccharide and flagellin. Beneficial commensal bacteria contain at least some TLR ligands [1], yet aberrant TLR stimulation by commensals may cause intestinal inflammation [4]. Therefore, mechanisms must be in place to allow the host to distinguish between pathogenic and commensal bacteria. Compartmentalization of TLRs facilitates this process. For example, TLR5, which recognizes flagellin, is only expressed on the basolateral side of intestinal epithelial cells, from which commensal bacteria are normally absent [5]. In contrast, pathogens possess virulence factors that allow them to penetrate into deeper tissues sites where receptors such as TLR5 can access them [1].

In addition to TLRs, there are innate immune sensors residing in the cytosol of mammalian cells that allow recognition of microbial molecules at this site or damage caused by pathogenic bacteria. One example is activation of the innate immune sensor Nod1 by the presence of bacterially-derived peptidoglycan [6]. For instance, Nod1 activation is triggered by the pathogen *Shigella flexneri*, which is thought to shed peptidoglycan during its residence in the host cytosol [7]. In addition, *Helicobacter pylori* may introduce peptidoglycan into host cells in a process dependent on a specialized secretion system, activating Nod1 [8]. Both of these microbial strategies, gaining entry into the host cytosol and utilizing a specialized secretion system, are thought to be more common among pathogens than commensals.

One such specialized secretion system found in a number of pathogenic bacteria is the type III secretion system (T3SS), which forms small pores in target host cells and delivers bacterial proteins into the host cytosol [9]. A common result of this “injection” is perturbation of normal host processes, to the benefit of the pathogen. One human pathogen that requires a T3SS for virulence is *Yersinia pseudotuberculosis*, which causes inflammation of the gastrointestinal tract exemplified by symptoms such as fever and swelling of gastric tissues and lymph nodes [10]. In healthy individuals, enteropathogenic yersiniosis is usually a self-limiting disease. However, in immunocompromised patients, the case fatality rate approaches 50% as a result of bacterial dissemination [11].

The *Y. pseudotuberculosis* T3SS is encoded on a virulence plasmid that is also found in the closely related human pathogens *Y. enterocolitica* and *Y. pestis* [12]. The *Yersinia* T3SS is composed of three protein subgroups: those that make up the injectisome, translocator Yops (Yersinia outer membrane proteins), and effector Yops. The injectisome is a needle-like structure that is evolutionarily related to the flagellar apparatus and has a central pore of about 20 Å [13],[14]. This needle apparatus is all that is required for secretion of the effector Yops, but is not sufficient for their translocation across the target cell plasma membrane. Targeting of effector Yops into the host cell cytosol requires the translocator proteins YopB, YopD, and LcrV, which are secreted through the T3SS apparatus and act to form channels in host cell membranes [15]. LcrV can be found associated with the tip of the needle apparatus [16] where it is thought to form a scaffold for the pore-forming proteins YopBD. T3SS effector Yops presumably travel through the type III needle and then through the pore made by YopBD in the host cell membrane. When the entire T3SS is functional, *Yersinia* translocate a group of five to six effector proteins into the host cytosol that interfere with target cell functions [17]. YopE, YopT, YopH, and YopO/YpkA target the host actin cytoskeleton, inhibiting phagocytosis and allowing the bacteria to remain largely extracellular. YopJ/YopP inhibits several inflammatory signaling pathways and influences the viability of a subset of host cells [18]–[20], while the function of YopM remains unknown [21].

The *Yersinia* T3SS pore, which forms during translocation of effector Yops, was recently suggested to trigger processing of the cytokines IL-1β and IL-18 in macrophages by the protease caspase-1 [22],[23]. Maturation of these cytokines has been linked to activation of a cytosolic innate immune complex called an inflammasome [24]. This type of complex is involved in detection of pore formation caused by a number of bacterial toxins [25]–[27]. Because other pathogens expressing specialized secretion systems, such as *H. pylori*, induce cytosolic innate immune sensors *distinct* from those found associated with inflammasomes [8],[28], we hypothesized that other host pathways may also be involved in recognizing the *Y. pseudotuberculosis* T3SS. Intriguingly, *Y. pseudotuberculosis* was previously reported to induce production of the chemokine IL-8 during infection of HeLa cells and this was dependent on expression of the *Y. pseudotuberculosis* T3SS [29]. Therefore, we tested the ability of several mammalian cell types to distinguish between *Y. pseudotuberculosis* expressing or lacking a functional T3SS. We show here that T3SS-competent *Y. pseudotuberculosis* triggers a TLR-independent transcriptional response that includes NFκB activation as well as induction of type I IFN-regulated genes, which are not known to be downstream of inflammasome activation. Furthermore, the NFκB pathway activated by the T3SS was independent of caspase-1-inflammasome activity. Recognition of T3SS-positive *Y. pseudotuberculosis* required bacterial expression of YopBD, but did not require any of the known effector Yops. Our results suggest that virulent *Yersinia* activate multiple cytosolic immune surveillance pathways as a consequence of utilizing a specialized secretion system.

## Results

### Macrophages produce TNF-α in response to extracellular *Y. pseudotuberculosis* expressing a functional T3SS independently of TLRs

To determine whether cytosolic innate immune sensors contribute to detection of *Yersinia*, an infection model of mammalian cells lacking TLR signaling was established. TLR activation generates strong innate immune responses, many of which overlap with those generated by cytosolic innate immune sensors [30]. In addition, *Yersinia* contain many TLR ligands. To circumvent this, we used bone marrow-derived macrophages (BMDMs) from mice that lack the two main TLR adaptor proteins, MyD88 and Trif, and hence lack TLR signaling (MyD88^−/−^/Trif^−/−^) [31]. The macrophage is a major in vivo target of *Yersinia* type III secretion [32], making it a good candidate for our experiments.

**Figure 1:**
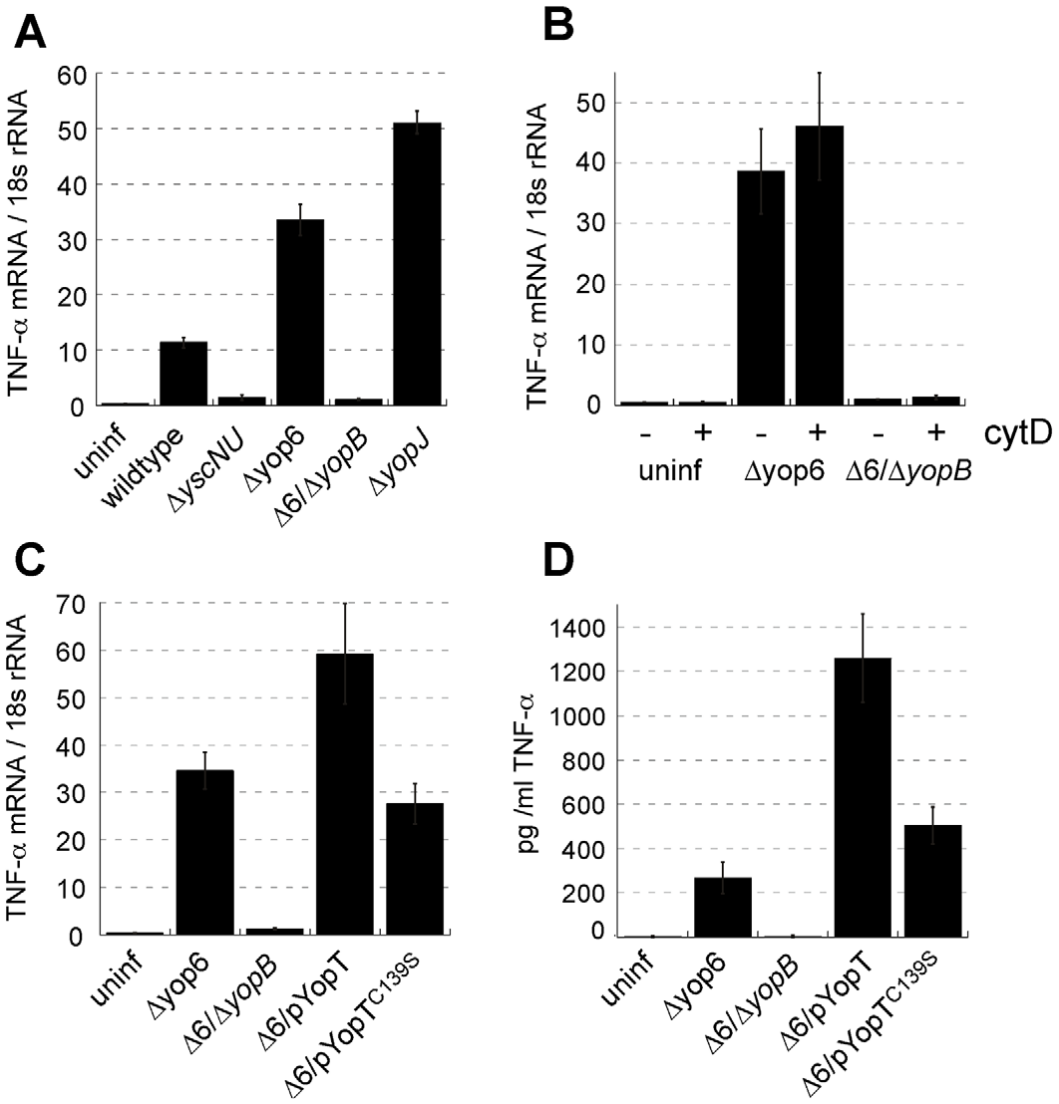
Extracellular *Y. pseudotuberculosis* expressing a functional T3SS translocator induces TLR-independent TNF-α production. MyD88^−/−^/Trif^−/−^ macrophages were infected with *Y. pseudotuberculosis* and *tnfa* mRNA levels (normalized to 18s rRNA) **(A–C)** and TNF-α protein levels **(D)** were quantified two hours post-inoculation. **(B)** Phagocytosis was inhibited by pre-incubating macrophages with cytochalasin D (cytD). The average of two to nine independent experiments ± standard error of the mean (sem) is shown.

We initially examined the production of the inflammatory cytokine TNF-α because it is produced downstream of several cytosolic innate immune sensors [28], but not following inflammasome activation [33],[34]. MyD88^−/−^/Trif^−/−^ BMDMs produced TNF-α mRNA in response to wildtype *Y. pseudotuberculosis*, but not to *Y. pseudotuberculosis* lacking a T3SS apparatus (Δ*yscNU*) (Table 1; Fig. 1A). This indicates that macrophages can distinguish *Yersinia* expressing a T3SS from one that lacks it.

**Table 1:** *Y. pseudotuberculosis* strains used in this study.

Strain	Background	Mutation	Reference
Wildtype	IP2666	Naturally lacks full length YopT	Bliska et al. 1991
Δyop6	IP2666	Δ*yopH*, Δ*yopE*, Δ*yopM*, Δ*yopO*, Δ*yopJ*	A. Davis and J. Mecsas (unpublished)
Δ6/Δ*yopB*	IP2666	Δ*yopH*, Δ*yopE*, Δ*yopM*, Δ*yopO*, Δ*yopJ*, Δ*yopB*	This work
Δ6/Δ*yopD*	IP2666	Δ*yopH*, Δ*yopE*, Δ*yopM*, Δ*yopO*, Δ*yopJ*, *yopD*ΔStyI	This work
Δ*yscNU*	IP2666	Deletion of yscNU operon	Balada-Llasat and Mecsas 2006
Δ*yopJ*	IP2666	Δ*yopJ*	Fisher et al. 2007
Δ6/pYopT	IP2666	Δ*yopH*, Δ*yopE*, Δ*yopM*, Δ*yopO*, Δ*yopJ*; pPHYopT	G. Viboud and J. Bliska (unpublished)
Δ6/pYopT^C139S^	IP2666	Δ*yopH*, Δ*yopE*, Δ*yopM*, Δ*yopO*, Δ*yopJ*; pPHYopTC139S	G. Viboud and J. Bliska (unpublished)
Δ6/Δ*yopN*	IP2666	Δ*yopH*, Δ*yopE*, Δ*yopM*, Δ*yopO*, Δ*yopJ*, Δ*yopN*	This work

To identify the component of the T3SS apparatus recognized by macrophages, we challenged MyD88^−/−^/Trif^−/−^ BMDMs with *Y. pseudotuberculosis* Δyop6 lacking all six known T3SS effector proteins. TNF-α was induced even more robustly (Fig. 1A), indicating that these effector proteins are not required for the macrophage response to the *Yersinia* T3SS. In contrast, *Y. pseudotuberculosis* Δ6/Δ*yopB* lacking the T3SS translocator component YopB did not induce any TNF-α in MyD88^−/−^/Trif^−/−^ BMDMs (Fig. 1A). In the absence of YopB, *Yersinia* cannot induce pore formation in target host cell membranes and cannot translocate any molecules into the host cell cytosol [35],[36]. However, the *Y. pseudotuberculosis* Δ6/Δ*yopB* strain does express a T3SS needle on its surface and can secrete the translocator component YopD under type III secretion-inducing conditions in vitro (Fig. S1A).

Since YopB was necessary for TLR-independent recognition of *Yersinia*, we reasoned that either the YopB protein itself was recognized by macrophages or that a YopB-mediated event, such as pore formation or translocation of an immune activating ligand into the host cytosol, was detected by macrophages. To distinguish between these possibilities, we assessed whether a strain of *Y. pseudotuberculosis* that was incapable of type III translocation, but still expressed YopB, could induce TLR-independent TNF-α production. A *Y. pseudotuberculosis* strain that carries a frameshift mutation in the translocator component YopD is unable to induce pore formation or effector protein translocation into target host cells [36], but can still secrete YopB into the culture supernatant (Fig. S1A). Similar to the strain lacking YopB, this YopD-deficient strain also did not induce TNF-α in the absence of TLR signaling (Fig. S1B). This indicates that rather than direct recognition of YopB, TLR-independent recognition of *Yersinia* expressing a functional T3SS involves detection of a YopBD-mediated event.

To test whether *Y. pseudotuberculosis* must be internalized in order for TLR-independent detection to occur, MyD88^−/−^/Trif^−/−^ BMDMs were preincubated with the actin cytoskeletal depolymerizing agent cytochalasin D (cytD) to inhibit phagocytosis. This treatment allowed 99% of the *Yersinia* to remain extracellular (data not shown). However, cytD treatment did not inhibit the TNF-α mRNA response of MyD88^−/−^/Trif^−/−^ BMDMs to *Yersinia* (Fig. 1B). This indicates that extracellular *Yersinia* expressing a functional T3SS can trigger TLR-independent innate immune signaling.

### T3SS effector proteins modulate the TLR-independent TNF-α response to *Y. pseudotuberculosis*

The T3SS effector protein YopJ has been shown to inhibit NFκB and MAP kinase signaling, leading to dampening of TLR-induced cytokine expression in cultured cells [18]. To determine if TLR-independent cytokine production is also inhibited by YopJ, we challenged MyD88^−/−^/Trif^−/−^ BMDMs with *Y. pseudotuberculosis* Δ*yopJ* (Fig. 1A). This strain induced an elevated level of TNF-α mRNA relative to the wildtype control, indicating that YopJ interferes with TLR-independent TNF-α production. Interestingly, *Y. pseudotuberculosis* Δyop6 lacking all six known T3SS effector proteins (Δyop6) induced TNF-α mRNA to a level three times greater than the wildtype *Y. pseudotuberculosis* strain, but 1.5-fold less than the Δ*yopJ Y. pseudotuberculosis* strain (p = 0.009; Fig. 1A). Therefore, in the absence of YopJ, other translocated effector proteins enhance the TLR-independent response to the T3SS. In fact, the presence of a single effector Yop was sufficient to recapitulate this stimulation. *Y. pseudotuberculosis* Δ6/pYopT expressing only the effector protein YopT induced 1.7-fold more TNF-α mRNA (p = 0.04; Fig. 1C) and five-fold more TNF-α protein (p = 0.008; Fig. 1D) than the *Y. pseudotuberculosis* Δyop6 strain. Furthermore, *Y. pseudotuberculosis* Δ6/pYopT^C139S^ expressing a catalytically inactive point mutant of YopT did not induce this enhanced TNF-α production (Fig. 1C–D). This indicates that while the effector protein YopJ partially inhibits TLR-independent TNF-α production triggered by *Yersinia*, the catalytic activity of YopT enhances TNF-α production.

### Macrophages launch a robust transcriptional response to T3SS translocator-positive *Yersinia* that includes NFκB- and type I IFN-regulated genes

The above results indicated that there may be a global response to the insertion of the *Y. pseudotuberculosis* T3SS into the host cell membrane. To identify the spectrum of host genes regulated in response to the *Y. pseudotuberculosis* T3SS, we determined the gene expression profile of MyD88^−/−^/Trif^−/−^ BMDMs after challenge with *Y. pseudotuberculosis* Δyop6 expressing a functional T3SS translocator compared to *Y. pseudotuberculosis* Δ6/Δ*yopB* expressing a T3SS needle but defective in type III translocation (Materials and Methods). We used strains lacking the known T3SS effector proteins to exclude macrophage genes controlled by the catalytic activity of these effectors. Even in the absence of the T3SS effector proteins, we found a large number of macrophage genes significantly regulated following challenge with *Y. pseudotuberculosis* compared to the uninfected condition (Fig. S2).

**Figure 2:**
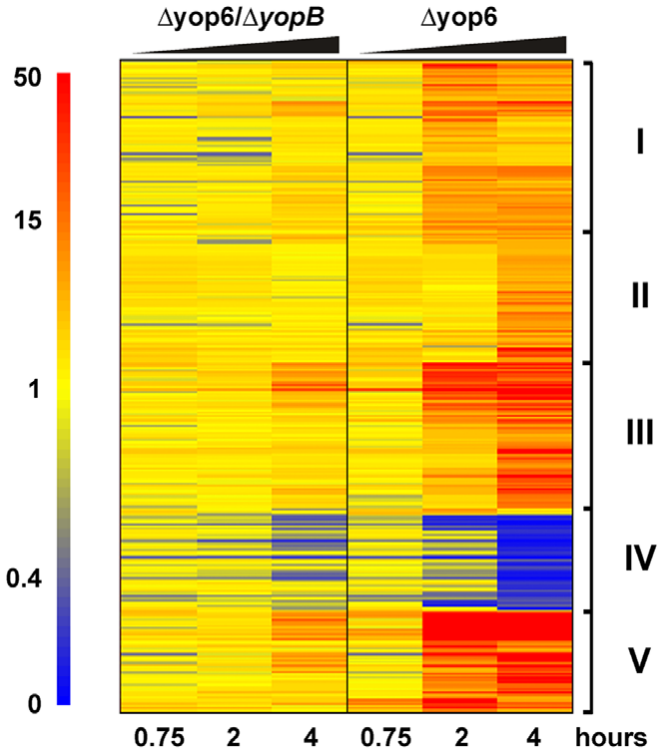
*Y. pseudotuberculosis* expressing a functional T3SS translocator induces robust macrophage gene expression. MyD88^−/−^/Trif^−/−^ macrophages were infected with *Y. pseudotuberculosis* Δyop6, Δ6/Δ*yopB*, or were left uninfected. Total RNA was isolated and Affymetrix GeneChip Mouse Genome 430 2.0 arrays were used to probe the relative gene expression profiles for each experimental condition. The expression of 266 murine genes (represented by 340 probe sets) was regulated by *Y. pseudotuberculosis* Δyop6 at least four-fold (p<0.05) more than by *Y. pseudotuberculosis* Δ6/Δ*yopB* during at least one time point. The probe sets were clustered into a heat map according to the Pearson correlation. From left to right, the 45 minute, two hour, and four hour time points are shown for each bacterial strain. The colorbar ranges from zero to 50-fold change in gene expression compared to the uninfected condition where red represents upregulated genes, yellow represents no change from uninfected, and blue represents downregulated genes.

**Figure 3:**
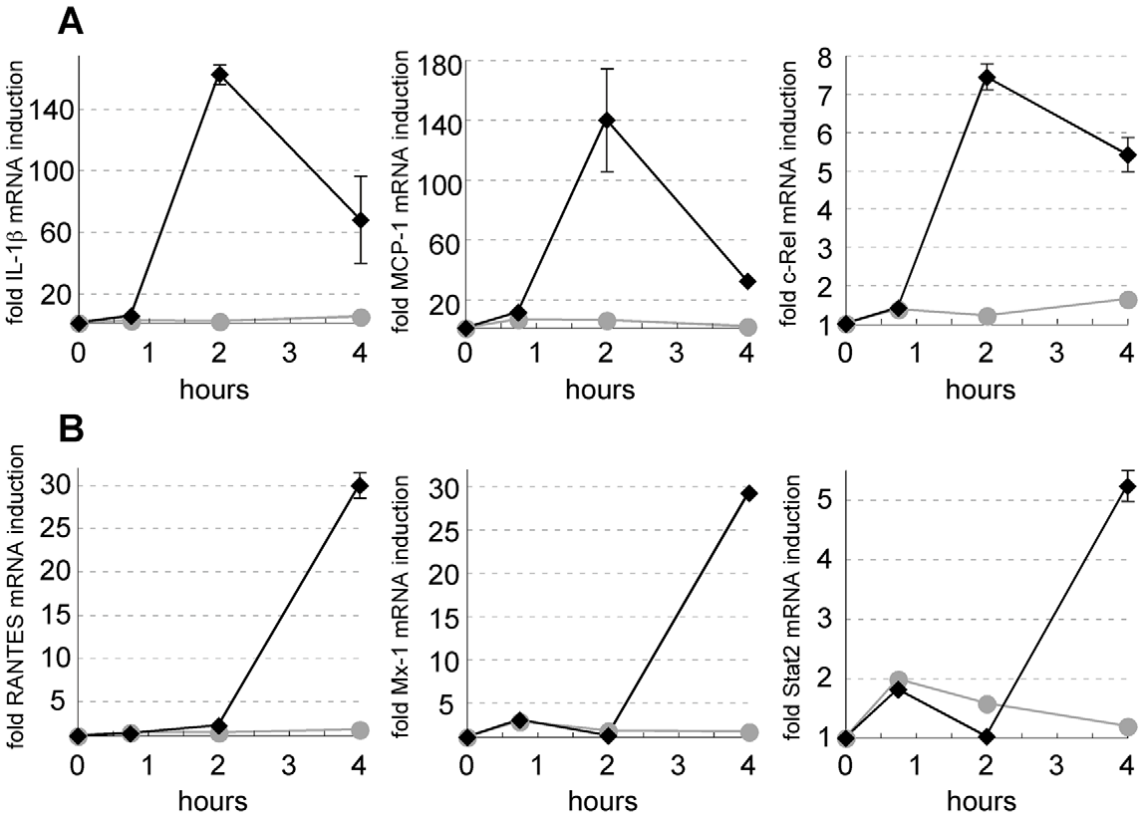
NFκB and type I IFN regulated genes exhibit different kinetic profiles following infection with T3SS translocator-positive *Y. pseudotuberculosis*. **(A)** Three NFκB-regulated genes and **(B)** three type I IFN-induced genes were selected from among the 266 genes identified by the microarray analysis described in Fig. 2. Shown is the average fold induction of the given probe set over the uninfected condition from *Y. pseudotuberculosis* Δyop6-infected (diamonds) and Δ6/Δ*yopB*-infected (circles) BMDMs ± sem.

The *Y. pseudotuberculosis* adhesin invasin has been reported to induce host cell signaling, consistent with the fact that it engages integrin receptors [37]. Because both the *Y. pseudotuberculosis* Δyop6 and Δ6/Δ*yopB* strains express invasin, integrin engagement may account for at least some of the alterations in host gene expression that are common to these two strains. Instead, we chose to focus on host genes that were differentially regulated in response to these two strains. Indeed, 266 genes were regulated by *Y. pseudotuberculosis* Δyop6 (compared to the uninfected condition) at least four-fold or more than by the Δ6/Δ*yopB* strain (Fig. 2; Datasets S1 and S2). These genes predominantly clustered into five groups based on their expression pattern (cluster I–V). Most of these genes were upregulated by the *Y. pseudotuberculosis* Δyop6 strain compared to the uninfected condition, although cluster IV contains downregulated genes. Other than TNF-α, a number of cytokines, chemokines, and costimulatory molecules were induced specifically in the presence of an intact T3SS translocator (Table 2). Furthermore, a number of genes involved in the NFκB signaling cascade were preferentially upregulated by *Y. pseudotuberculosis* Δyop6 compared to the Δ6/Δ*yopB* strain (Table 3). There was a similar pattern of expression for many of the genes controlled by this transcription factor, as transcription was induced by two hours post-inoculation with the *Y. pseudotuberculosis* Δyop6 strain and then subsequently tapered (Fig. 3A shows three examples). This was similar to the TNF-α expression pattern as determined by quantitative PCR analysis (qPCR; Fig. S3). Consistent with the panel of genes that were identified in this fashion, NFκB could indeed be preferentially activated by *Y. pseudotuberculosis* Δyop6 compared to *Y. pseudotuberculosis* Δ6/Δ*yopB*, based on monitoring infected 293T cells expressing a NFκB luciferase reporter (see below).

**Table 2:** Cytokine and chemokine genes induced preferentially by *Y. pseudotuberculosis* expressing an intact T3SS translocator.

Gene name^1^	Description	Cluster
Mip1a/Ccl3	Macrophage inflammatory protein 1-alpha	I
IL-15	Interleukin 15	II
IL-15Rα	Interleukin 15 receptor, alpha	II
Mip2/Cxcl2	Macrophage inflammatory protein 2	III
IP10/Cxcl10	IFN-inducible protein of 10 kDa	III
RANTES/Ccl5	Regulated upon Activation, Normal T-cell Expressed, and Secreted	III
TNFsf4	Tumor necrosis factor (ligand) superfamily, member 4	III
Mip1β/Ccl4	Macrophage inflammatory protein 1-beta	III
KC/Cxcl1	Neutrophil-activating protein 3	V
IL-1α	Interleukin 1-alpha	V
IL-1β	Interleukin 1-beta	V
IL-10	Interleukin 10	V
Tnfsf9	Tumor necrosis factor (ligand) superfamily, member 9	V
Tnfsf14	Tumor necrosis factor (ligand) superfamily, member 14	V
MCP-1/Ccl2	Monocyte chemoattractant protein 1	V
MCP-3/Ccl7	Monocyte chemoattractant protein 3	V
Bsf3/Clcf1	B-cell stimulating factor 3	V

1 Genes listed were hand-selected following literature review of genes listed in Datasets S1 and S2, which are those that were upregulated at least 4-fold more by *Y. pseudotuberculosis* Δyop6 compared to *Y. pseudotuberculosis* Δ6/ΔyopB (p<0.05) according to microarray analysis.

**Table 3:** NFκB cascade genes induced preferentially by *Y. pseudotuberculosis* expressing an intact T3SS translocator.

Gene name^1^	Description	Cluster
IκBζ/Nfkbiz	Nuclear factor of kappa light polypeptide gene enhancer in B-cells inhibitor, zeta	I
c-Rel	Reticuloendotheliosis oncogene; NFκB subunit	I
Rip2/RICK	Receptor-interacting protein 2	I
Tank	TRAF family member-associated NFκB activator	I
Traf5	TNF receptor-associated factor 5	II
Nfkbie	Nuclear factor of kappa light polypeptide gene enhancer in B-cells inhibitor, epsilon	III
Nfkbie	Nuclear factor of kappa light polypeptide gene enhancer in B-cells inhibitor, epsilon	III
Tnfaip3/A20	Tumor necrosis factor, alpha-induced protein 3	III
Traf1	TNF receptor-associated factor 1	V

1 Genes listed were hand-selected following literature review of genes listed in Datasets S1 and S2, which are those that were upregulated at least 4-fold more by *Y. pseudotuberculosis* Δyop6 than by *Y. pseudotuberculosis* Δ6/ΔyopB (p<0.05) according to microarray analysis.

A subset of genes represented in the heatmap in Fig. 2 are known to be controlled by cytokines of the type I interferon (IFN) family and had a distinct expression pattern. These genes were not induced significantly until four hours post-inoculation with the *Y. pseudotuberculosis* Δyop6 strain (Fig. 3B shows three examples). This was consistent with the induction pattern of the cytokine IFN-inducible protein of 10 kDa (IP10) that we obtained using qPCR (Fig. S4A). In addition, most of the type I IFN-inducible genes identified by microarray analysis fell into cluster II, where expression was highest at four hours post-inoculation (Fig. 2; Table 4). Consistent with induction of type I IFN-regulated genes at later time points, mRNA levels of the gene encoding the type I IFN family member IFNβ were significantly upregulated (p<0.0001) over uninfected levels by *Y. pseudotuberculosis* Δyop6 (5.4-fold±0.9) compared to the Δ6/Δ*yopB* strain (1.2-fold±0.2) two hours post-inoculation (Fig. S4B). Similar to TNF-α, the observed induction of IFN-β by *Y. pseudotuberculosis* Δyop6 did not depend on the bacteria being internalized into macrophages because the level of IFNβ mRNA was not reduced in the presence of cytochalasin D (data not shown).

**Table 4:** Type I IFN-inducible genes induced preferentially by *Y. pseudotuberculosis* expressing an intact T3SS translocator.

Gene name^1^	Description	Cluster
Irf1	Interferon regulatory factor 1	I
Ifit1/Ifi56	Interferon-induced protein with tetratricopeptide repeats 1	II
Ifit2/Ifi54	Interferon-induced protein with tetratricopeptide repeats 2	II
Ifit3/Ifi49	Interferon-induced protein with tetratricopeptide repeats 3	II
Mx1	Myxovirus resistance protein 1	II
Mx2	Myxovirus resistance protein 2	II
Gbp2	Interferon-induced guanylate-binding protein 2	II
Ifi203	Interferon-inducible protein 203	II
RIG-I	Retinoic acid-inducible gene 1	II
Oasl1	2′–5′ oligoadenylate synthetase-like 1	II
Stat2	Signal transducer and activator of transcription 2	II
IP10/Cxcl10	IFN-inducible protein of 10 kDa	III
RANTES/Ccl5	Regulated upon Activation, Normal T-cell Expressed, and Secreted	III
Ifi202b	Interferon-inducible protein 203b	V

1 Genes listed were hand-selected following literature review of genes listed in Datasets S1 and S2, which are those that were upregulated at least 4-fold more by *Y. pseudotuberculosis* Δyop6 than by *Y. pseudotuberculosis* Δ6/ΔyopB (p<0.05) according to microarray analysis.

**Figure 4:**
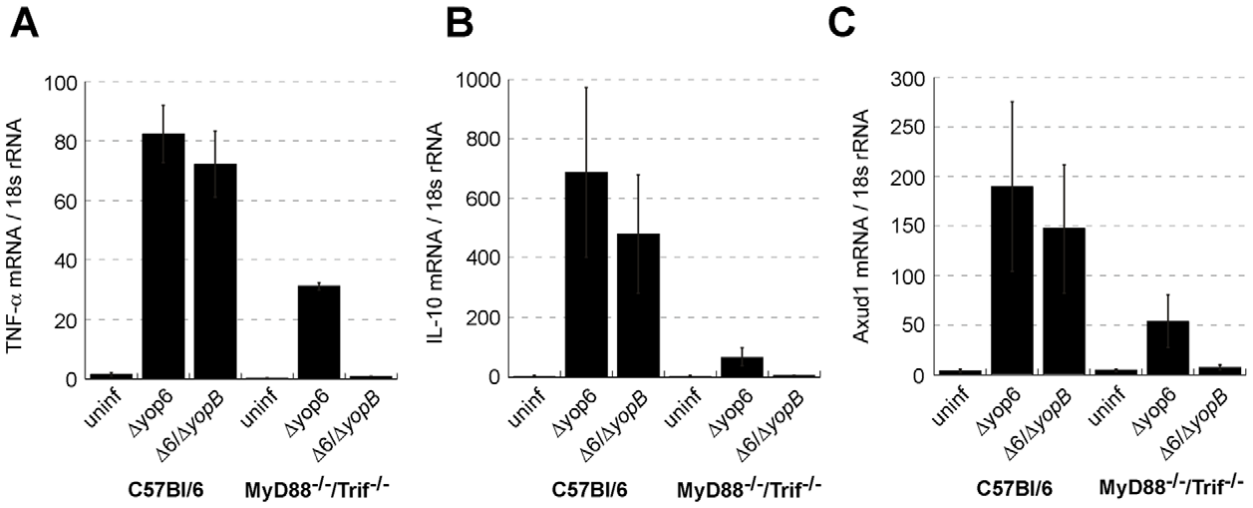
T3SS translocator-positive and –negative *Y. pseudotuberculosis* can be differentiated by a TLR-independent recognition pathway. C57Bl/6 and MyD88^−/−^/Trif^−/−^ macrophages were infected with *Y. pseudotuberculosis* and total RNA isolated at 2 hours post-inoculation. **(A)** *tnfa*, **(B)** *il10*, and **(C)** *axud1* mRNA levels were quantified (normalized to 18s rRNA). Data represents average±sem from three independent experiments.

**Figure 5:**
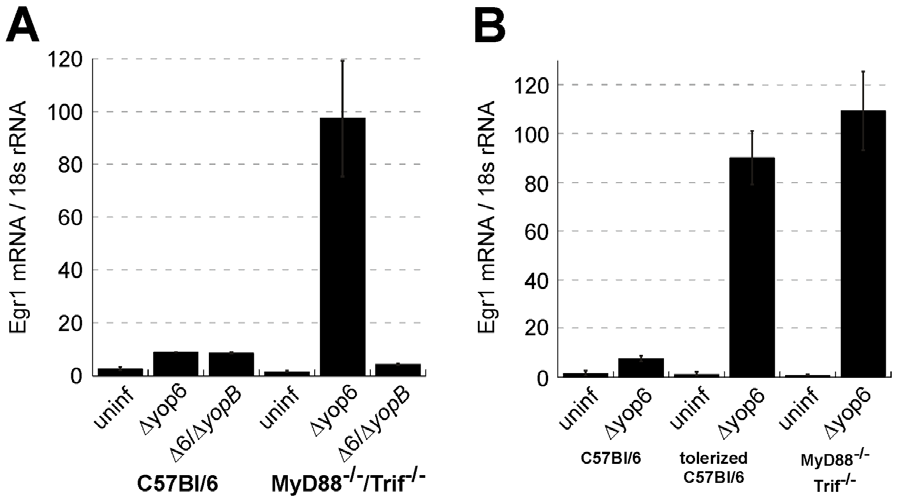
Tolerization of TLR signaling in wildtype macrophages relieves *egr1* repression during infection with *Y. pseudotuberculosis*. C57Bl/6 and MyD88^−/−^/Trif^−/−^ macrophages were **(A)** infected with *Y. pseudotuberculosis* and total RNA isolated at 2 hours post-inoculation or **(B)** were first incubated with heat-killed *Y. pseudotuberculosis* overnight (tolerized) or were left untreated. The tolerized or untreated macrophages were then infected with live *Y. pseudotuberculosis* Δyop6 and total RNA isolated two hours post-inoculation. *Egr1* mRNA levels (normalized to 18s rRNA) were quantified. Data represents average ± sem from two independent experiments.

To validate some of our microarray results, we used qPCR to verify that a subset of the genes identified in our microarray analysis were indeed induced by *Y. pseudotuberculosis* Δyop6 independently of TLRs. We infected MyD88^−/−^/Trif^−/−^ BMDMs with *Y. pseudotuberculosis* Δyop6 or Δ6/Δ*yopB* and analyzed mRNA levels of select genes two hours post-inoculation. We found that, similar to TNF-α, genes encoding the anti-inflammatory cytokine IL-10, the cysteine- and serine-rich nuclear protein Axud1, and the transcription factor early growth response protein 1 (Egr1) were transcribed by macrophages in response to *Y. pseudotuberculosis* Δyop6 but not to the Δ6/Δ*yopB* strain (Fig. 4; Fig. 5A). These results support those obtained from our microarray analysis.

To determine if the presence of TLR signaling alters the expression of genes induced in response to an intact T3SS translocator, wildtype C57Bl/6 macrophages were challenged with *Y. pseudotuberculosis* Δyop6 or Δ6/Δ*yopB*. In the presence of TLR signaling, macrophages transcribed the genes encoding TNF-α, IL-10, and Axud1 in response to both *Y. pseudotuberculosis* strains (Fig. 4). This indicates that TLR ligands, such as LPS, trigger macrophage transcription of these genes independently of the T3SS.

### TLRs antagonize induction of a subset of genes that respond to a functional T3SS translocator

The *egr1* and *egr2* genes were induced preferentially by the T3SS translocator-positive *Y. pseudotuberculosis* Δyop6 strain in macrophages lacking TLR signaling (Fig. 5A and microarray data not shown). However, while *egr1* was induced in wildtype macrophages in response to both *Y. pseudotuberculosis* Δyop6 and Δ6/Δ*yopB*, the mRNA levels were 11-fold lower than that induced by the Δyop6 strain in MyD88^−/−^/Trif^−/−^ BMDMs. This indicates that TLR signaling may negatively regulate *egr1* expression. In order to test this hypothesis, we tolerized wildtype macrophages to TLR ligands by incubating them overnight with a low level of heat-killed *Y. pseudotuberculosis*. This type of treatment has been used previously to dampen subsequent TLR responses [38]. We then challenged these tolerized macrophages with live *Y. pseudotuberculosis* Δyop6 and measured Egr1 mRNA levels. While untolerized wildtype macrophages produced only low levels of Egr1 mRNA, tolerized wildtype macrophages produced Erg1 mRNA levels similar to that produced by MyD88^−/−^/Trif^−/−^ BMDMs (Fig. 5B). Similar results were obtained for Egr2 (data not shown). Tolerization treatment of MyD88^−/−^/Trif^−/−^ BMDMs did not affect Egr1 expression (data not shown). These data suggest that cross-talk exists between the TLR-dependent and -independent signaling pathways, modulating the response of a subset of genes to the T3SS.

### The *Y. pseudotuberculosis* T3SS activates NFκB independently of the cytosolic peptidoglycan sensors Nod1 and Nod2

**Figure 6:**
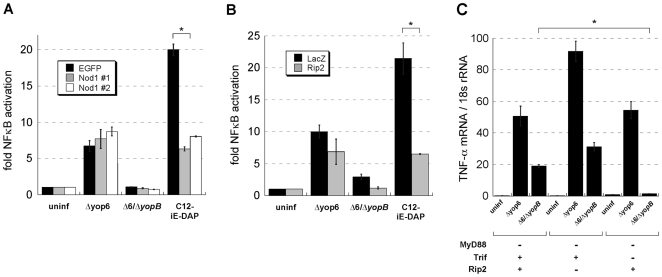
*Y. pseudotuberculosis* triggers NFκB activation independently of Nod1 and Nod2. **(A–B)** 293T cells were transfected with plasmids encoding siRNA against Nod1 or EGFP **(A)** or Rip2 or LacZ **(B)** as well as a plasmid expressing an NFκB luciferase reporter gene. The transfected cells were then infected with *Y. pseudotuberculosis* or treated with a synthetic Nod1 ligand, C12-iE-DAP, that is able to access the cell cytosol. Four hours post-inoculation or post-treatment, NFκB activation was quantified by measuring luminescence. Data shown is the average fold NFκB activation (over uninfected/untreated) ± sem from one independent, representative experiment and each experiment was repeated for a total of three (Nod1) to four (Rip2) replicates. * Statistically significant decrease in C12-iE-DAP-induced NFκB activation according to the student t-Test. P<0.01 for Nod1 shRNA compared to EGFP control. P<0.03 for Rip2 shRNA compared to LacZ control. There was no statistical difference in NFκB activation induced by *Y. pseudotuberculosis* Δyop6 with either Nod1 (p>0.2) or Rip2 (p>0.3) knockdown. **(C)** MyD88^−/−^, MyD88^−/−^/Rip2^−/−^, and MyD88^−/−^/Trif^−/−^ macrophages were infected with *Y. pseudotuberculosis* Δyop6 or Δ6/Δ*yopB* and *tnfa* mRNA levels (normalized to 18s rRNA) were measured two hours post-inoculation. Data shown is the average ± sem from one independent, representative experiment, which was repeated for a total of two replicates. * The only statistically significant decrease in *tnfa* mRNA levels compared to the Trif^+/+^/Rip2^+/+^ control cells was observed for the *Y. pseudotuberculosis* Δ6/Δ*yopB* strain in Trif^−/−^/Rip2^+/+^ cells (student t-Test, p<0.002).

The cytoplasmic innate immune sensors Nod1 and Nod2, which are activated by peptidoglycan moieties, have been shown to activate NFκB [6]. Rip2 is a kinase that is downstream of both Nod1 and Nod2 and is required for induction of genes that respond to these sensors [39],[40]. We hypothesized that Nod1 and/or Nod2 could be involved in NFκB activation in response to *Yersinia* infection, as peptidoglycan fragments could have access to the host cell cytosol via the T3SS. To test this, we knocked down expression of Nod1 and Rip2 in 293T cells using shRNAs and monitored NFκB activation using a luciferase reporter. We found that NFκB-driven luciferase expression in 293T cells could be triggered by an acylated derivative of the Nod1 ligand iE-DAP (C12-iE-DAP), which can enter the cell cytosol when added exogenously to the cell culture (Fig. 6AB). Compared to a control shRNA, shRNA against Nod1 (Fig. 6A) or Rip2 (Fig. 6B) caused a clear decrease in C12-iE-DAP-induced NFκB activation. However, knocking down either Nod1 or Rip2 did not significantly decrease NFκB activation induced by the *Y. pseudotuberculosis* Δyop6 strain. In fact, in two out of four experiments the level of NFκB activation increased slightly upon *Y. pseudotuberculosis* Δyop6 infection during Rip2 knockdown compared to the LacZ shRNA control (data not shown).

To gain further evidence that the NFκB signaling observed in response to *Y. pseudotuberculosis* was independent of a peptidoglycan activation pathway, BMDMs from MyD88^−/−^, MyD88^−/−^/Rip2^−/−^, and MyD88^−/−^/Trif^−/−^ mice were analyzed. TNF-α mRNA levels were determined after challenge of BMDMs with either *Y. pseudotuberculosis* Δyop6 or Δ6/Δ*yopB* in order to analyze activation of an NFκB-dependent gene in the absence of MyD88 signaling (Fig. 6C). Rip2 was dispensable for signaling downstream of both *Y. pseudotuberculosis* strains, regardless of the presence or absence of a functional T3SS translocator (Fig. 6C, MyD88^−/−^ compared to MyD88^−/−^/Rip2^−/−^ cells). In fact, MyD88^−/−^/Rip2^−/−^ cells produced somewhat more TNF-α mRNA in response to either strain of *Y. pseudotuberculosis* compared to MyD88^−/−^/Rip2^+/+^ strains. In the case of the bacterial strain lacking the T3SS translocator (Δ6/Δ*yopB*), however, TNF-α signaling appeared to be solely TLR-dependent, as Trif was necessary for signaling in the absence of MyD88 (Fig. 6C, MyD88^−/−^ compared to MyD88^−/−^/Trif^−/−^ cells). In contrast, T3SS translocator-dependent signaling occurred in BMDMs from all the relevant mouse genotypes, regardless of intact TLR or Rip2 signaling. Therefore, we conclude that NFκB activation that occurs in response to T3SS translocator-positive *Y. pseudotuberculosis* does not involve Nod1 or Nod2 signaling.

### The *Y. pseudotuberculosis* T3SS induces secretion of TNF-α, but not IL-1β, independently of caspase-1

T3SS-positive strains of *Yersinia* have been shown to trigger maturation and secretion of the inflammatory cytokine IL-1β in a manner that is dependent on the host protease caspase-1 [22],[41]. To determine whether caspase-1 is also involved in NFκB signaling triggered by T3SS translocator-positive *Y. pseudotuberculosis*, we infected MyD88^−/−^/Trif^−/−^ macrophages with *Y. pseudotuberculosis* in the presence or absence of the caspase-1 inhibitor Z-YVAD(OMe)-FMK, and measured levels of secreted TNF-α (Table 5). TNF-α levels remained unchanged in the presence of caspase-1 inhibitor. This was in contrast to IL-1β secretion, which was inhibited by Z-YVAD(OMe)-FMK by three-fold (p<0.01). These data indicate that, as previously reported, the *Yersinia* T3SS induces host caspase-1 activation and IL-1β release [22],[41]. However, the *Yersinia* T3SS also induces host production and secretion of TNF-α and the signaling pathway responsible is independent of caspase-1.

**Table 5:** T3SS translocator-positive *Y. pseudotuberculosis* induces TNF-α, but not IL-1β, production independently of the inflammasome component caspase-1.

Treatment	IL-1β (pg/ml)	TNF-α (pg/ml)
None	1.3±1.5	0.8±0.2
Δyop6^1^	113.0±7.6	1260.4±71.9
Δyop6+YVAD^2^	37.5±8.3^3^	1295.5±84.3
Δ6/Δ*yopB*	1.5±0.6	6.0±0.8

1 MyD88^−/−^/Trif^−/−^ BMDMs were infected with *Y. pseudotuberculosis* Δyop6 or Δ6/Δ*yopB* and supernatant IL-1β and TNF-α levels measured four hours post-inoculation.

2 Two hours prior to infection, the caspase-1 inhibitor Z-YVAD(OMe)-FMK was added to the BMDMs.

3 Statistically significant decrease in IL-1β concentration compared to the Δyop6-infected, no YVAD condition (Students t-Test; p<0.01).

### Type III secreted products introduced into the host cytosol independently of the T3SS apparatus do not stimulate cytokine production

**Figure 7:**
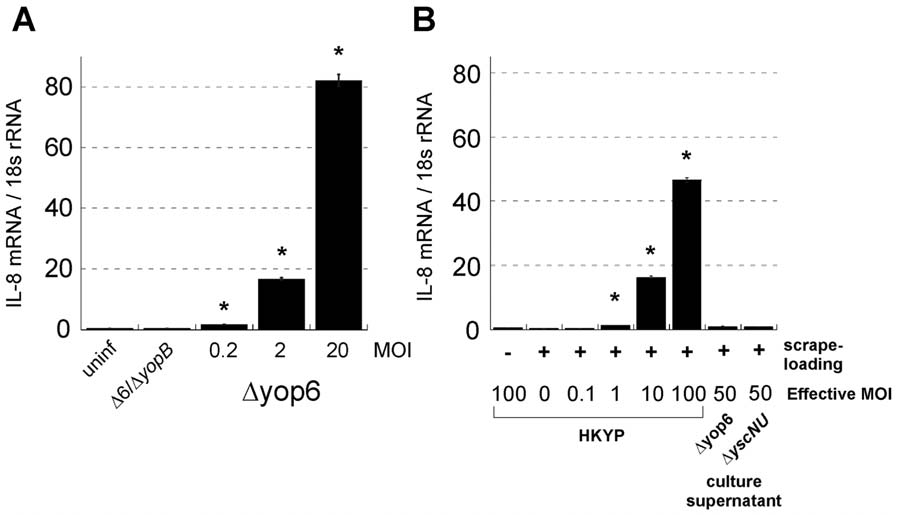
Neither introduction of type III secreted molecules into the cytosol of 293T cells nor nonspecific membrane disruption induce IL-8 production. **(A)** 293T cells were infected with different MOIs of live *Y. pseudotuberculosis* for two hours. **(B)** Filtrate from *Y. pseudotuberculosis* Δyop6 or Δ*yscNU* culture grown under type III secretion-inducing conditions was scrape-loaded into 293T cells for 5–10 minutes and the scrape-loaded cells incubated for an additional two hours. The Δ*yscNU* strain does not express a T3SS, does not secrete any T3SS cargo into the culture supernatant, and is the negative control for scrape-loading Δyop6 culture filtrate. Alternatively, varying amounts of heat-killed and lysed *Y. pseudotuberculosis* or buffer were scrape-loaded into 293T cells for 5–10 minutes and the scrape-loaded cells incubated for an additional two hours. Total RNA was isolated and *il8* mRNA levels (normalized to 18s rRNA) were quantified. Data shown is the average±sem from one independent, representative experiment and each experiment was repeated for a total of two **(A)** to three **(B)** replicates. * Statistically significant increase (p<0.0001) in *il8* levels according to the student t-Test compared to uninfected **(A)** or scrape only **(B)** controls.

T3SS translocator-dependent cytokine induction may stem from recognition of a YopBD pore or from introduction of innate immune stimulating molecules into the host cytosol. To distinguish between these two possibilities, we set up two systems in which products secreted by *Y. pseudotuberculosis* via the T3SS were introduced into the host cytosol independently of the YopBD pore, using either scrape-loading [42] or transfection. 293T cells were chosen for scrape-loading because they form loose adhesions to the extracellular matrix and have a high survival rate after scrape loading (unpublished observations). To determine the sensitivity of the 293T cell response to T3SS translocator-positive *Yersinia*, we infected 293T cells with varying numbers of live *Y. pseudotuberculosis* and measured induction of the NFκB-regulated cytokine IL-8. An inoculum of as little as one *Y. pseudotuberculosis* Δyop6 per five 293T cells (multiplicity of infection, MOI = 0.2) induced IL-8 mRNA production after two hours (Fig. 7A). In contrast, even a MOI = 20 of *Y. pseudotuberculosis* Δ6/Δ*yopB* did not induce IL-8. These results indicate that 293T cells produce detectable IL-8 levels in response to a small number of *Yersinia* expressing a functional T3SS translocator.

To test specifically whether molecules secreted via the *Y. pseudotuberculosis* T3SS could induce cytokine production in the absence of YopBD-mediated pore formation, we collected culture supernatant from *Y. pseudotuberculosis* grown under type III secretion-inducing conditions and introduced it into 293T cells via scrape-loading (see Materials and Methods). Under *in vitro* type III secretion-inducing conditions, *Y. pseudotuberculosis* strains that express a T3SS needle apparatus secrete proteins and possibly other molecules into the supernatant (see Fig. S1A). Scrape-loading of culture supernatant from *Y. pseudotuberculosis* Δyop6, equivalent to that produced by 50 bacteria per 293T cell, did not result in an increase in IL-8 mRNA (Fig. 7B). In contrast, lysate from heat-killed *Y. pseudotuberculosis* (HKYP), which contains molecules such as peptidoglycan known to induce cytosolic innate immune signaling, triggered IL-8 production when delivered inside 293T cells via scrape loading (Fig. 7B). This was achieved even when the equivalent of one HKYP was scrape-loaded into one 293T cell. However, even the equivalent of 100 HKYP per 293T cell was insufficient to induce IL-8 production in the absence of scrape-loading. Importantly, scrape-loading alone (i.e.-nonspecific membrane disruption) did not trigger IL-8 production.

To support these results, we used transfection of primary macrophages as a second method to deliver culture supernatants into the host cell cytosol independently of the YopBD pore. In order to demonstrate the sensitivity of the macrophages to T3SS translocator-positive *Y. pseudotuberculosis*, we challenged macrophages with varying numbers of *Y. pseudotuberculosis* Δyop6. A MOI = 0.1 (for TNF-α) or a MOI = 1.0 (for IFNβ) induced detectable cytokine mRNA levels (Fig. S5A,D). To test specifically whether molecules secreted by the T3SS could induce cytokine production in the absence of YopBD-mediated pore formation, we transfected MyD88^−/−^/Trif^−/−^ macrophages with supernatants from bacterial cultures grown under type III secretion-inducing conditions. Neither TNF-α nor IFNβ was detected even after transfection with supernatant equivalent to about 70 *Y. pseudotuberculosis* per macrophage (Fig. S5B,E). In contrast, transfection of small amounts of HKYP lysate into macrophages elicited both a significant TNF-α and IFNβ response (Fig. S5C,F).

Collectively, these data indicate that neither introduction of *Y. pseudotuberculosis* type III secreted products into the cytosol of mammalian cells independently of the YopBD pore nor nonspecific membrane disruption can recapitulate induction of NFκB-regulated cytokines or type I IFN triggered by *Y. pseudotuberculosis* expressing a functional T3SS translocator.

## Discussion

We have characterized a previously unknown mammalian innate immune response to the enteropathogen *Y. pseudotuberculosis*. Unlike TLR recognition of *Yersinia*, the TLR-, Nod1/2-, and caspase-1-independent pathway described here is able to distinguish between *Y. pseudotuberculosis* expressing a functional T3SS and *Y. pseudotuberculosis* lacking this essential virulence determinant. Because the ensuing immune response includes at least two distinct signaling branches with different expression profiles, we propose the involvement of multiple cytosolic immune sensors. Bacterial uptake into host cells was not required for T3SS-stimulated NFκB activation or type I IFN production, indicating that extracellular *Yersinia* capable of type III translocation can activate diverse cytosolic innate immune signaling pathways. Although the host response that is detailed here does not require the presence of known T3SS effectors, cytokine production triggered by these recognition events is modulated in opposing directions by at least two such effector proteins, YopJ and YopT. Collectively, these results suggest multiple layers of host manipulation carried out by the *Y. pseudotuberculosis* T3SS.

Dozens of animal and plant pathogens use specialized secretion systems to deliver bacterial proteins into the cytosol of target host cells in order to manipulate host functions and promote virulence [9],[43]. We have shown here that the mammalian innate immune system possesses the ability to specifically recognize and respond to pathogenic *Y. pseudotuberculosis* based on bacterial utilization of one such virulence-associated secretion system. We hypothesize that different features of the *Y. pseudotuberculosis* secretion system are recognized by distinct cytosolic innate immune receptors, launching a unique immune response.

Inflammasome complexes, which contain such cytosolic sensors, are involved in caspase-1 activation, facilitating release of the cytokines IL-18 and IL-1β in response to *Y. pseudotuberculosis*, *Y. pestis*, *Pseudomonas aeruginosa*, *Salmonella typhimurium*, *Legionella pneumophila*, and *Burkholderia pseudomallei* that express functional T3/4SSs [22], [44]–[52]. Many of these inflammasome activation events also require bacterial flagellin, suggesting that flagellin is delivered into the cytosol of target host cells through these specialized secretion systems [53], triggering an immune response. While *Y. pestis* encodes an inactive *flhD* allele that results in lack of flagellin expression, T3SS-competent *Y. pestis* still activates caspase-1 [22], perhaps because the pore-forming ability of the *Yersinia* T3SS is sufficient to stimulate inflammasome activity. Such a mechanism of stimulation would be similar to the response of inflammasome complexes to a diverse set of pore-forming toxins [25]. Interestingly, when the inactive, *Y. pestis* allele of *flhD* is crossed into the *Y. pseudotuberculosis* Δyop6 background, TNF-α is still induced in a TLR-independent manner (data not shown). This suggests that NFκB activation triggered by the *Yersinia* T3SS is also independent of flagellin. Furthermore, we could find no role for the inflammasome-associated cytosolic sensor Nalp3 in *Y. pseudotuberculosis*-induced TNF-α production. In addition, the potassium ionophore nigericin, which induces IL-1β secretion via the Nalp3 inflammasome, did not promote TNF-α production (data not shown). Lastly, we found that NFκB induced by T3SS-positive *Y. pseudotuberculosis* is independent of caspase-1, ruling out a role for caspase-1-inflammasome complexes in this pathway (Table 5).

*Y. pseudotuberculosis* was previously shown to induce production of the NFκB-regulated chemokine IL-8 in HeLa cells and this was dependent on YopB expression [29]. Here we show that in addition to stimulation of IL-8 production in 293T cells (Fig. 7) and HeLa cells [29], T3SS-competent *Y. pseudotuberculosis* triggers a large-scale transcriptional response in mouse macrophages. This represents a much broader response to a T3SS than previously appreciated. Interestingly, T3SS-competent *Citrobacter rodentium* has been shown to induce IL-8 and TNF-α production in HT29 human intestinal epithelial cells dependent on the peptidoglycan sensors Nod1 and Nod2, respectively [28]. We also observed *Y. pseudotuberculosis*-induced IL-8 production in HT29 cells dependent on an intact T3SS (data not shown). However, we did not find a role for Nod1 or Nod2 in T3SS-dependent NFκB activation in our 293T cell reporter system or in primary macrophages (Fig. 6). Similarly, T3SS-competent *B. pseudomallei* triggers IL-8 production in 293T cells and this response is also independent of Nod1 [54]. The nature of the putative ligand(s) that triggers NFκB-dependent cytokine production by T3SS-competent *Y. pseudotuberculosis* and *B. pseudomallei* remains to be determined. Intriguingly, induction of IL-8 by *B. pseudomallei* does not occur in the absence of a functional T3SS even though this bacterium is able to access the host cytosol [54],[55]. This indicates that the presence of a bacterium in the host cell cytosol does not necessarily lead to immune detection. Rather, it is the active process of type III translocation into host cells that allows recognition to occur.

In contrast to innate immune ligands such as peptidoglycan, microbially-derived nucleic acids induce a distinct mammalian immune response that includes type I IFN family members [56]. Expression of these cytokines involves not only NFκB, but also the transcription factor IRF3 [57]. Following production and secretion of type I IFNs, the type I IFN receptor becomes activated in a paracrine and autocrine manner. This leads to the upregulation of a number of type I IFN-responsive genes, such as the chemokine IP10. Both cytosolic RNA and DNA can induce this type of amplified type I IFN response [56],[58]. Specific RNA moieties are recognized by two sensors, Mda5 and RIG-I, that reside in the mammalian cytosol [59]. However, the identity of the cytosolic DNA sensors remain unclear. The protein DAI/DLM-1/ZBP1 has shown to be involved in upregulation of type I IFN in response to DNA in some cell types but not in others [60]–[62], suggesting possible redundancy with other, as yet unidentified receptors. Indeed, several cell types from DAI^−/−^ mice still responded to cytosolic DNA by producing type I IFN [63]. Another cytosolic DNA receptor, AIM2, has been described that induces NFκB and caspase-1 activation, but not type I IFN [64]–[67]. While at least some DNA detection pathways are independent of the RNA sensors RIG-I and Mda5, another pathway exists that depends on RNA polymerase III-mediated transcription of cytosolic DNA templates, leading to synthesis of RNA intermediates that trigger RIG-I-dependent type I IFN production [68],[69]. Interestingly, over 60% of the genes induced by introducing DNA into mouse embryonic fibroblasts [70] were also regulated by T3SS translocator-positive *Yersinia* in MyD88^−/−^/Trif^−/−^ macrophages (data not shown). It is tempting to speculate that nucleic acids may enter the host cell cytosol during *Y. pseudotuberculosis* type III translocation, triggering a type I IFN response.

The T4SSs of two other pathogens, *Brucella abortus* and *L. pneumophila*, have been implicated in induction of type I IFNs [71],[72]. Because T4SSs are evolutionarily related to conjugation machinery, it is feasible that DNA is translocated into the target host cell, activating a cytosolic innate immune sensor. However, the nature of the *Y. pseudotuberculosis* type I IFN-inducing ligand is unclear, as no evidence exists that nucleic acids are secreted or translocated through a T3SS. Interestingly, we found that adding exogenous, synthetic RNA to MyD88^−/−^/Trif^−/−^ macrophages infected with *Y. pseudotuberculosis* led to an enhanced IFNβ response (Fig. S6). Importantly, this was dependent on the bacteria expressing a functional T3SS translocator, indicating that extracellular nucleic acids can leak into the cytosol of mammalian cells during the process of type III translocation.

While an extracellular pathogen, group B streptococcus, was recently shown to trigger type I IFN production in a TLR-independent manner, bacterial phagocytosis and phagosomal membrane disruption were required [73]. To our knowledge, the *Yersinia* T3SS-dependent induction of a type I IFN response shown here is the first demonstration of an extracellular pathogen inducing TLR-independent type I IFN in the absence of bacterial internalization into host cells. This indicates that *Y. pseudotuberculosis* may employ a novel mechanism of innate immune activation. Whether type I IFN induction by the T3SS hinders or aids *Y. pseudotuberculosis* survival in vivo remains to be determined since the type I IFN receptor has been shown to facilitate or inhibit bacterial growth in mice depending on the type of pathogen used [74]–[78]. It will be important to determine the contribution of T3SS-induced type I IFN signaling to survival of mice during infection with *Yersinia*.

Previous work has shown that the *Yersinia* T3SS effector protein YopJ inhibits the NFκB, MAP kinase, and IRF3 signaling pathways in target host cells [79]. Consistent with these data, we observed reduced TNF-α levels triggered by *Y. pseudotuberculosis* expressing YopJ. Surprisingly, we also found that *Y. pseudotuberculosis* expressing either the T3SS effector protein YopT (Fig. 1) or YopE (data not shown) induced elevated TNF-α levels. This was dependent on the catalytic activity of these proteins because YopT and YopE point mutants that lack a catalytically active residue did not trigger these enhanced TNF-α levels (Fig. 1 and data not shown). YopT is a cysteine protease that cleaves the prenyl group of RhoGTPases, mislocalizing them from the plasma membrane [80]. YopE is a Rho GTPase activating protein (GAP) that accelerates the hydrolysis of GTP on RhoGTPases [17]. Both YopT and YopE lead to a loss of activated RhoGTPases from the plasma membrane and to actin cytoskeletal rearrangements. Interestingly, the RhoGTPase Rac1 was recently shown to negatively regulate Nod2 signaling, possibly by altering its subcellular localization [81],[82]. In addition, proteins that modulate the actin cytoskeleton have been linked to Nod1-dependent activation of NFκB [83]. While we could not find a role for Nod1 or Nod2 in *Yersinia*-induced NFκB activation (Fig. 6), it is possible that RhoGTPases may affect the activity of other cytosolic innate immune sensors.

**Figure 8:**
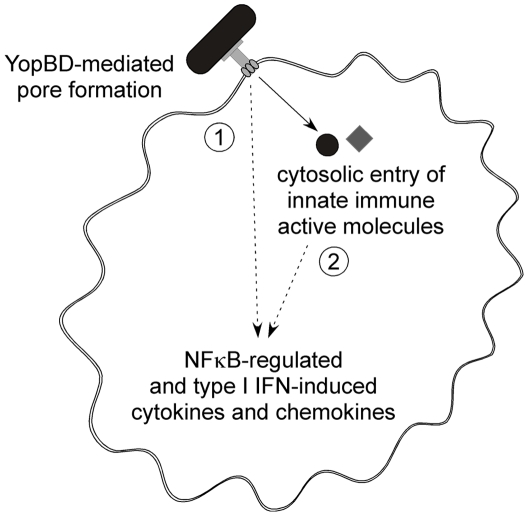
Activation of cytosolic innate immune signaling by the *Y. pseudotuberculosis* T3SS requires a YopBD-mediated translocation event. *Y. pseudotuberculosis* expressing a functional T3SS translocator triggers NFκB and type I IFN innate immune signaling independently of T3SS effector proteins. This response requires the pore-forming proteins YopBD and cannot be recapitulated by nonspecific membrane disruption nor by introducing *Y. pseudotuberculosis* T3SS secreted molecules into host cells independently of the YopBD pore. We propose that both (1) YopBD-mediated membrane insertion or pore formation and (2) cytosolic entry of multiple immune activating molecules capable of triggering different transcriptional events are required for full activation of the innate immune response to the *Y. pseudotuberculosis* T3SS.

Based on the data presented here, we propose that extracellular *Y. pseudotuberculosis* activates the cytosolic innate immune system during the process of type III translocation in a manner that is dependent on both YopBD-mediated pore formation as well as entrance of innate immune activating molecules into the target host cell cytosol (Fig. 8). The ability of extracellular *Yersinia* to trigger this response indicates that phagosomal degradation of the bacteria is not required to release the active ligands. In addition, the diverse transcriptional response to T3SS translocator-positive *Yersinia* in the absence of TLR signaling points to involvement of cytosolic innate immune sensors since no other known surface-associated proteins are capable of inducing such a robust, *de novo* response [84]–[86]. If the putative receptor(s) recognizing T3SS-competent *Yersinia* are not surface-associated, then the active components must penetrate the host cell to trigger the observed signaling. It is possible that the *Yersinia* T3SS induces the opening of a surface channel that allows an immune activating ligand to enter the cytosol. Alternatively, YopBD-mediated pore formation may allow molecules other than T3SS effector proteins inside target host cells. A possible model is that more than one such molecule enters the host cell, explaining the varied nature of the innate immune response. However, it is possible that one molecule is responsible for initiating the entire response and that its cognate receptor is capable of triggering several different signaling pathways with distinct kinetics. We could not recapitulate the signaling triggered by T3SS-competent *Yersinia* by introducing type III secreted molecules into host cells independently of the T3SS nor by inducing nonspecific membrane disruption. It is possible that the immune activating ligands are not secreted via the *Yersinia* T3SS during growth in bacterial culture media, but are translocated into host cells. Alternatively, the act of translocation (YopBD-mediated entry of activating ligands) may be specifically required. The nature of these putative ligands remains unclear, as we did not find a role for peptidoglycan or flagellin in innate immune recognition of the *Yersinia* T3SS.

The data presented here describing induction of inflammatory cytokines in response to T3SS translocator-positive *Y. pseudotuberculosis* is consistent with the well-established ability of enteropathogenic *Yersinia* to cause acute, localized gut inflammation [10]. Our results demonstrating cross-talk between TLR signaling and T3SS-dependent cytosolic signaling in response to *Yersinia* (Fig. 5) indicate that both pathways contribute to the overall immune response observed during *Yersinia* infection. Furthermore, in cells that are normally unresponsive to some TLR ligands (such as intestinal epithelial cells), recognition of a functional T3SS may play a primary role in specifically recognizing pathogenic bacteria. It will be important to determine the specific contribution of TLR-independent recognition of *Yersinia* in terms of both virulence of the pathogen as well as the intestinal inflammation caused by infection.

## Materials and Methods

All animal use procedures were in strict accordance with the NIH Guide for the Care and Use of Laboratory Animals and were approved by the Tufts University Institutional Animal Care and Use Committee.

### Bacterial strains

The *Y. pseudotuberculosis* strains used in this study (Table 1) were derived from the serogroup III strain IP2666pIB1 (Bliska et al. PNAS 1991). The Δyop6 and Δ6/Δ*yopB* strains were constructed using the suicide plasmids described by Logsdon and Mecsas [87]. The Δ6/Δ*yopN* strain was constructed using the suicide plasmid described by Davis and Mecsas [88]. The Δ6/pYopT and Δ6/pYopT^C139S^ strains were constructed by electroporating pPHYopT and mating pPHYopTC139S, kind gifts from Dr. James Bliska, into the Δyop6 strain. These plasmids express wildtype YopT or YopT carrying a substitution mutation in a catalytically active residue under the control of the YopH promoter. The Δ6/pYopT strain was able to cause rounding of macrophages (indicative of Yop intoxication) and caused the entry of Rac1 into the nucleus of Cos1 cells (data not shown), while the Δ6/pYopT^C139S^ strain did not. The Δ6/*yopD*ΔstyI strain was constructed by mating pSB890 encoding the *yopD*ΔstyI allele, a kind gift from Dr. Tessa Bergsbaken and Dr. Brad Cookson, into the Δyop6 strain. The *yopD*ΔstyI allele was constructed according to the strategy described by Viboud et al. [29] and harbors a frameshift mutation in *yopD*. *Y. pseudotuberculosis* strains were grown in 2× YT overnight at 26°C with agitation for incubation with macrophages or 293T cells. The overnight cultures were diluted into 2× YT containing 20 mM sodium oxalate and 20 mM MgCl_2_ (low calcium medium) in order to obtain an OD_600_ of 0.2. These back-diluted cultures were grown at 26°C for 1.5 h with agitation, and transferred to 37°C for an additional 1.5 h with agitation to induce the expression of the type III secretion system [89]. In order to make culture fitrates for scrape-loading, the low calcium cultures were filtered through a 0.22 µm syringe filter (Millipore). The filtrate was then either left at its original concentration or was concentrated two-fold in a speed-vac centrifuge.

### Mice

MyD88^−/−^/Trif^−/−^ mice were a gift from Dr. Shizuo Akira. C57Bl/6 mice were purchased from Jackson Laboratories. MyD88^−/−^ [90] and Rip2^−/−^ [39] mice have been described.

### Primary macrophages and cell lines

Bone marrow derived macrophages (BMDMs) were obtained as previously described [74], except engineered NIH-3T3 cells were used as a source of CSF [38]. The harvested macrophages were frozen and stored in liquid nitrogen. Frozen aliquots were thawed and plated one day prior to use in Dulbecco's modified Eagle medium (Gibco) supplemented with 10% fetal bovine serum (HyClone) and L-glutamine (Gibco). 293T cells were passaged and plated in the identical medium.

### Macrophage infections

BMDMs were plated onto six-well plates (Falcon) at a density of 10^6^ cells per ml and incubated at 37° with 5% CO_2_ overnight. One day later, the cells were inoculated with approximately 1–3×10^7^ *Y. pseudotuberculosis* grown under type III secretion-inducing conditions (see Bacterial Strains section above) for a multiplicity of infection (MOI) of approximately 10∶1 (unless otherwise specified). The bacteria were then allowed to settle onto the macrophage monolayer for 30 minutes at 37° with 5% CO_2_. This treatment allowed approximately two-thirds of the macrophages to be associated with one or two bacteria thirty minutes post-inoculation (data not shown). The media was then filtered through a 0.22 µm syringe filter (Millipore). This was done in order to remove any non-attached or non-internalized bacteria without removing any cytokines secreted by the macrophages during the initial 30 minutes of incubation. This treatment prevented the macrophage monolayer from becoming overwhelmed with bacteria by the end of the inoculation period and yet allowed any extracellular bacteria attached to macrophages and in the process of type III secretion to remain viable. Two hours post-inoculation, macrophage supernatants were collected and stored at −80°C. Macrophage monolayers were then washed once with phosphate-buffered saline and total RNA was harvested using the RNAqueous kit (Ambion) according to the manufacturer's instructions. For inhibition of phagocytosis, BMDMs were treated with 2 µM cytochalasin D (Sigma) 30 minutes prior to infection.

### Quantitative PCR

The DNA-free kit (Ambion) was used to remove any contaminating genomic DNA from the total RNA samples harvested as described above. Total RNA yield was calculated using a NanoDrop ND-1000 spectrophotometer (Thermo Scientific) and 2 µg RNA was used to make cDNA as previously described [74]. SYBR Green PCR master mix (Applied Biosystems) was used for qPCR reactions according to the manufacturer's instructions using a Mx3005P (Stratagene) or DNA Engine Opticon 2 (BioRad) qPCR machine and a 60°C annealing temperature. The results were analyzed using the Mx3005P or Opticon 2 software. QPCR primers used in this study are described in Table S1. All qPCR primers were validated in silico using NCBI mapviewer (www.ncbi.nlm.nih.gov/projects/mapview/).

### TCA precipitation

*Y. pseudotuberculosis* was grown under type III secretion inducing conditions as described in the Bacterial Strains section above, except the cultures were incubated at 37°C for an additional 30 minutes (two hours total). The cultures were plated on LB plates to determine CFU/ml. 900 µl of culture was pelleted and 800 µl supernatant was removed. Trichloroacetic acid was added to the supernatant for a final concentration of 10% and the mixture was incubated on ice for 15 minutes. The mixture was pelleted for 15 minutes at 13,000 rpm and the pellet was washed with acetone. The washed pellet was resuspended in 50 µl Laemmli buffer and frozen at −80°C. The samples were thawed and boiled for five minutes. Approximately half of each sample (the samples were normalized for CFU/ml) was run on a 12.5% polyacrylamide gel and the protein bands visualized with coomassie blue staining.

### Microarray

MyD88^−/−^/Trif/^−/−^ BMDMs were infected as described above with *Y. pseudotuberculosis* Δyop6 or Δ6/Δ*yopB* or were left uninfected. After 45 minutes, two hours, or four hours post-inoculation, total RNA was harvested as described above except that two wells of macrophages were pooled per condition to yield enough RNA for microarray analysis. The infection was then repeated on a separate day to yield two biological replicates per condition. 5 µg total RNA was used to make probes for GeneChip Mouse Genome 430 2.0 arrays using the One-Cycle cDNA synthesis protocol and array hybridization was performed according to the manufacturer's instructions (Affymetrix). A GeneChip Fluidics Station was used to wash the arrays and a GeneChip Scanner was used to read the arrays (Affymetrix). GeneChip Operating Software was used to analyze the quality of the hybridizations (Affymetrix). GeneSpring GX software (Agilent) was used to analyze the microarray data and the GC Robust Multiarray Averaging method was used for data normalization.

### Heat-killed *Y. pseudotuberculosis*

To make heat-killed *Y. pseudotuberculosis*, the wildtype IP2666 strain was grown overnight at 26° with agitation in Luria-Bertani broth. The overnight culture was heat-killed at 60°C for one hour, aliquoted, and frozen at −80°C in the absence of glycerol. Aliquots were thawed at room temperature before use. Very few intact bacteria could be visualized by microscopy (unpublished observations), indicating lysis occurred upon freeze/thawing. The culture was plated before and after heat-killing to calculate the live colony forming unit (CFU) equivalents in the heat-killed mixture and to ensure complete killing.

### Tolerization of TLR signaling

C57Bl/6 BMDMs were plated onto six-well plates (Falcon) at a density of 2×10^6^ per well in 2 ml of media containing 2×10^5^ heat-killed *Y. pseudotuberculosis*. The cells were incubated overnight at 37° with 5% CO_2_. The next day, the tolerized macrophages were inoculated with live *Y. pseudotuberculosis* or left uninfected as described in the Macrophage infections section above.

### Nod1 and Rip2 knockdown

293T cells were plated at a density of 2.5×10^4^ per 100 µl in 96 well plates (Corning). One day later, the cells were transfected with 400 ng per well of plasmid encoding shRNA specific for Nod1, Rip2 or control shRNA (EGFP for Nod1 and LacZ for Rip2) using Lipofectamine 2000 (Invitrogen) according to the manufacturer's instructions. The Nod1 and EGFP shRNA constructs were cloned into the pRNAT-U6.1/Neo vector backbone (GenScript). The Nod1 shRNA construct #1 and shRNA EGFP control were based on previously published siRNA sequences (Viala et al. 2004). The Nod1 shRNA construct #2 (5′- GATCCCGTCAAAGGCAGCACGGAAGTGCTTGATATCCGGCACTTCCGTGCTGCCTTTGATTTTTTCCAAA -3′) was designed using GenScript siRNA Design Center (V. Losick and R. Isberg). The Rip2 and LacZ shRNA plasmids were purchased from InvivoGen. 200 ng per well of an NFκB luciferase reporter plasmid (Stratagene) was transfected into 293T cells either simultaneously with the Nod1 or EGFP shRNA plasmids or 24 hours after the Rip2 or LacZ shRNA plasmids. After 48 hours of shRNA plasmid transfection, the 293T cells were infected with 3–4×10^5^ *Y. pseudotuberculosis* Δyop6 or Δ6/Δ*yopB* per well. Since the 293T cells doubled at least twice during the 48 hour transfection period, the MOI was approximately 3∶1. Alternatively, transfected 293T cells were treated with 1 µg/ml C12-iE-DAP, an acylated derivative of the iE-DAP Nod1 ligand that is able to enter the cytosol of cultured cells (InvivoGen). After four hours of infection with *Y. pseudotuberculosis* or treatment with C12-iE-DAP, luminescence was measured using the SteadyLite Plus reporter gene assay system (PerkinElmer) and a SpectraMax M5 microplate reader (Molecular Devices).

### Caspase-1 inhibition

BMDMs were plated onto 24 well plates (Falcon) at a concentration of 5.75×10^5^ per ml and incubated overnight. The irreversible caspase-1 inhibitor Z-YVAD(OMe)-FMK (Santa Cruz Biotechnology) was added at a concentration of 20 µM two hours prior to infection. The cells were inoculated with approximately 1–4×10^7^ *Y. pseudotuberculosis* grown under type III secretion-inducing conditions (see Bacterial Strains section above) for a multiplicity of infection (MOI) of approximately 20∶1. Four hours post-inoculation, supernatants were collected and frozen at −80°C.

### TNF-α and IL-1β protein detection

For Fig. 1D, the amount of TNF-α protein in supernatants of infected macrophages was measured as described in Auerbuch et al. [74] using the Cytometric Bead Array Mouse Inflammation Kit (BD Biosciences). For Table 5, frozen supernatants were centrifuged at 13,000 rpm for 1 min and TNF-α and IL-1β protein levels were measured using a plate-based ELISA (eBioscience) and a Victor 3 microplate reader (PerkinElmer).

### Scrape-loading

The scrape-loading protocol used was adapted from McNeil et al. [42]. 293T cells were plated at a density of 0.5–1×10^6^ cells per ml in six well plates. One day later, the cells were washed once with warm HBSS. 300 µl warm HBSS plus ligand were added to each well. MOI equivalents of 0.1–100 HKYP or 150 µl of culture filtrate from live *Y. pseudotuberculosis* grown under type III secretion-inducing conditions (see Bacterial Strains section above) were used as ligands. Cell monolayers were then scraped with a cell scraper and incubated at 37°C for five to ten minutes. The cells were then washed off the plastic with 5 ml of warm HBSS and pelleted. The cells were resuspended in 2 ml warm media, plated on fresh six well plates, and incubated at 37°C for two hours. Alternatively, 293T cells were incubated with HKYP (MOI 100) in the absence of scraping for two hours as a negative control. Total RNA was then harvested as described for infected macrophage monolayers and IL-8 mRNA levels measured by qPCR as described above.

### 293T cell infections

293T cells were plated at a density of 5×10^5^ cells per ml in six well plates. One day later, the cells were inoculated with *Y. pseudotuberculosis* grown under type III secretion-inducing conditions at a MOI of 1∶10 to 10∶1 (*Yersinia*:293Ts). The monolayer supernatants were filtered after 30 minutes as described for macrophage infections. IL-8 mRNA was measured after two hours of total infection as described above.

### Macrophage transfection

Primary macrophages were plated onto six well plates at a concentration of 10^6^ per ml. One day later, MOI equivalents of 0.1–10 HKYP or 250 µl per well of culture filtrate from live *Y. pseudotuberculosis* grown under type III secretion-inducing conditions were transfected into the macrophages using Lipofectamine 2000 (Invitrogen) according to the manufacturer's recommendations (5 µl Lipofectamine 2000 reagent per well). The cells were then incubated for two hours. Total RNA was harvested and *tnfa* or *ifnb* mRNA levels measured as described above. The culture fitrates did not inhibit the transfection process because transfection of both the synthetic RNA poly(I:C) and culture filtrate together into macrophages did not lead to reduced *ifnb* mRNA levels compared to poly(I:C) alone (data not shown).

## Supporting Information

Table S1 Quantitative PCR primers used in this study.
(0.03 MB DOC)

Figure S1 *Y. pseudotuberculosis* lacking YopD expression secretes YopB but does not induce TLR-independent TNF-α production. (A) Supernatants from *Y. pseudotuberculosis* cultures grown under type III secretion-inducing conditions were subject to TCA precipitation, run on a polyacrylamide gel, and stained with coomassie blue. (B) MyD88^−/−^/Trif^−/−^ macrophages were infected with *Y. pseudotuberculosis* and *tnfa* mRNA levels (normalized to 18s rRNA) were quantified two hours post-inoculation.
(9.69 MB TIF)

Figure S2 *Y. pseudotuberculosis* expressing a functional T3SS translocator regulates a greater number of macrophage genes than translocator-negative *Y. pseudotuberculosis*. MyD88^−/−^/Trif^−/−^ macrophages were infected with *Y. pseudotuberculosis* Δyop6 or Δyop6/Δ*yopB* or were left uninfected. Total RNA was isolated 45 minutes, two hours, or four hours post-inoculation and Affymetrix GeneChip Mouse Genome 430 2.0 arrays were used to probe the relative gene expression profiles for each experimental condition. Probe sets regulated by either *Y. pseudotuberculosis* Δyop6 (diamonds) or *Y. pseudotuberculosis* Δyop6/Δ*yopB* (x) at least two-fold over the uninfected condition (p<0.1) were selected. Shown are the log_10_ of the fold-change values of these selected probe sets. Positive values indicate up-regulation and negative values indicate down-regulation compared to the uninfected condition. For the 45 minute time point, N = 280 (Δyop6) and N = 263 (Δyop6/Δ*yopB*). For the two hour time point, N = 1248 (Δyop6) and N = 232 (Δyop6/Δ*yopB*). For the four hour time point, N = 2215 (Δyop6) and N = 338 (Δyop6/Δ*yopB*).
(0.17 MB PDF)

Figure S3 *Tnfa* mRNA levels peak two hours after inoculation with translocator-positive *Y. pseudotuberculosis*. MyD88^−/−^/Trif^−/−^ macrophages were infected with *Y. pseudotuberculosis* Δyop6 (diamonds) or Δyop6/Δ*yopB* (circles) and total RNA isolated at 2 hours, 4 hours, or 6 hours post-inoculation. Average *tnfa* mRNA levels (normalized to 18s rRNA) are shown.
(0.10 MB PDF)

Figure S4 *Ifnb* mRNA and type I IFN-regulated gene expression are preferentially induced by T3SS translocator-positive *Y. pseudotuberculosis*. MyD88^−/−^/Trif^−/−^ macrophages were infected with *Y. pseudotuberculosis* Δyop6 (diamonds) or Δyop6/Δ*yopB* (circles) and total RNA isolated at 2 hours (A and B), 4 hours, or 6 hours (A) post-inoculation and qPCR analysis performed. Average *ip10* (A) and *ifnb* (B) mRNA levels (normalized to 18s rRNA) are shown ± sem.
(0.11 MB PDF)

Figure S5 Transfection of type III secreted molecules into primary macrophages does not induce TNF-α or IFNβ. (A,D) MyD88^−/−^/Trif^−/−^ macrophages were infected with varying amounts of live *Y. pseudotuberculosis* for two hours. Filtrates from cultures of *Y. pseudotuberculosis* grown under type III secretion-inducing conditions (B,E) or HKYP (C,F) were transfected into MyD88^−/−^/Trif^−/−^ macrophages and incubated for two hours. Total RNA was isolated and *tnfa* (A–C) and *ifnb* (D–F) mRNA levels (normalized to 18s rRNA) were quantified. Data shown is the average ± sem from one independent, representative experiment and each experiment was repeated for a total of two replicates. * Statistically significant increase (p<0.0001) in cytokine mRNA levels according to the student t-Test compared to uninfected (A) or lipofectamine only (C,F) controls. ** p = 0.0001. *** p<0.0005. *Y. pseudotuberculosis* strains lacking the regulatory protein YopN hypersecrete Yops [86] and the Δ6/Δ*yopN* strain was included to test whether T3SS cargo from a hypersecreting *Y. pseudotuberculosis* strain could trigger IL-8 production.
(0.15 MB PDF)

Figure S6 Exogenously-added, synthetic RNA synergizes with *yopB*-expressing, but not *yopB*-deficient, *Y. pseudotuberculosis* to induce IFNβ mRNA expression. MyD88^−/−^/Trif^−/−^ macrophages were infected with *Y. pseudotuberculosis* Δyop6, Δyop6/Δ*yopB*, or Δ*yopJ* in the presence or absence of 1µg/ml poly(I:C). Total RNA was isolated at 2 hours post-inoculation and qPCR analysis performed. Data shown is the average *ifnb* mRNA level (normalized to 18s rRNA)±sem from one independent, representative experiment and the experiment was repeated for a total of three replicates.
(0.10 MB PDF)

Dataset S1 Identity of genes regulated by *Y. pseudotuberculosis* Δyop6 at least four-fold (p<0.05) more than by *Y. pseudotuberculosis* Δyop6/Δ*yopB*. Fold-change compared to the uninfected condition and the p value for each probe set is shown.
(0.11 MB XLS)

Dataset S2 Identity of genes found in clusters I–V shown in the heatmap in Figure 2A.
(0.11 MB XLS)
