# Supplementary material for: Innate Immune Recognition of Yersinia pseudotuberculosis Type III Secretion
Source: PLoS Pathog. 2009 Dec 4;5(12):e1000686. doi: 10.1371/journal.ppat.1000686 (PMC2779593; doi:10.1371/journal.ppat.1000686)
Supplement: Table S1 — Quantitative PCR primers used in this study. (0.03 MB DOC) [file ppat.1000686.s001.doc]

**Supplementary Table 1.** Quantitative PCR primers used in this study.

|  | **Species** | **Forward** | **Reverse** | Reference |
| --- | --- | --- | --- | --- |
| TNFα | mouse | gcaccaccatcaaggactcaa | tcgaggctccagtgaattcg | Auerbuch et al. 2004 |
| IFNβ  (pair 1) | mouse | TTCTCCACCACAGCCCTCTC | CTTTCCATTCAGCTGCTCCAG | Auerbuch et al. 2004 |
| IFNβ  (pair 2) | mouse | AAGAGTTACACTGCCTTTGCCATC | CACTGTCTGCTGGTGGAGTTCATC | Roth-Cross et al. (Weiss) 2007 JI |
| IP10 | mouse | aaatcatccctgcgagcctat | tcgtggcaatgatctcaacac | McCaffrey and Portnoy (unpublished) |
| Egr1 | mouse | GGGAGAGGCAGGAAAGACATAA | TCTGAGATCTTCCATCTGACCTAAGA | Baron et al (Mercola) 2003 Oncogene |
| IL-10 | mouse | ACCTGGTAGAAGTGATGCCCCAGGCA | CTATGCAGTTGATGAAGATGTCAAA | Ehlers et al 1992 JI |
| Axud1 | mouse | GAGCACTTGCTTCCTGGAGT | TCACAAAGAGCAGCAACAGG | Araki et al. 2006 Brain Research |
| IL-8 | human | AATCTGGCAACCCTAGTCTGCTA | AAACCAAGGCACAGTGGAACA | Nazarenko et al 2002 Nucleic Acids Research |
| 18s | mouse | CGCCGCTAGAGGTGAAATTCT | CATTCTTGGCAAATGCTTTCG | Totemeyer et al. 2005 I&I 73:3 |
| 18s | human | GACTCATTGGCCCTGTAATTGGAATGAGTC | CCAAGATCCAACTACGAGCTT | Nazarenko et al 2002 Nucleic Acids Research |
