# Supplementary figures and images for: Innate Immune Recognition of Yersinia pseudotuberculosis Type III Secretion
Source: PLoS Pathog. 2009 Dec 4;5(12):e1000686. doi: 10.1371/journal.ppat.1000686 (PMC2779593; doi:10.1371/journal.ppat.1000686)

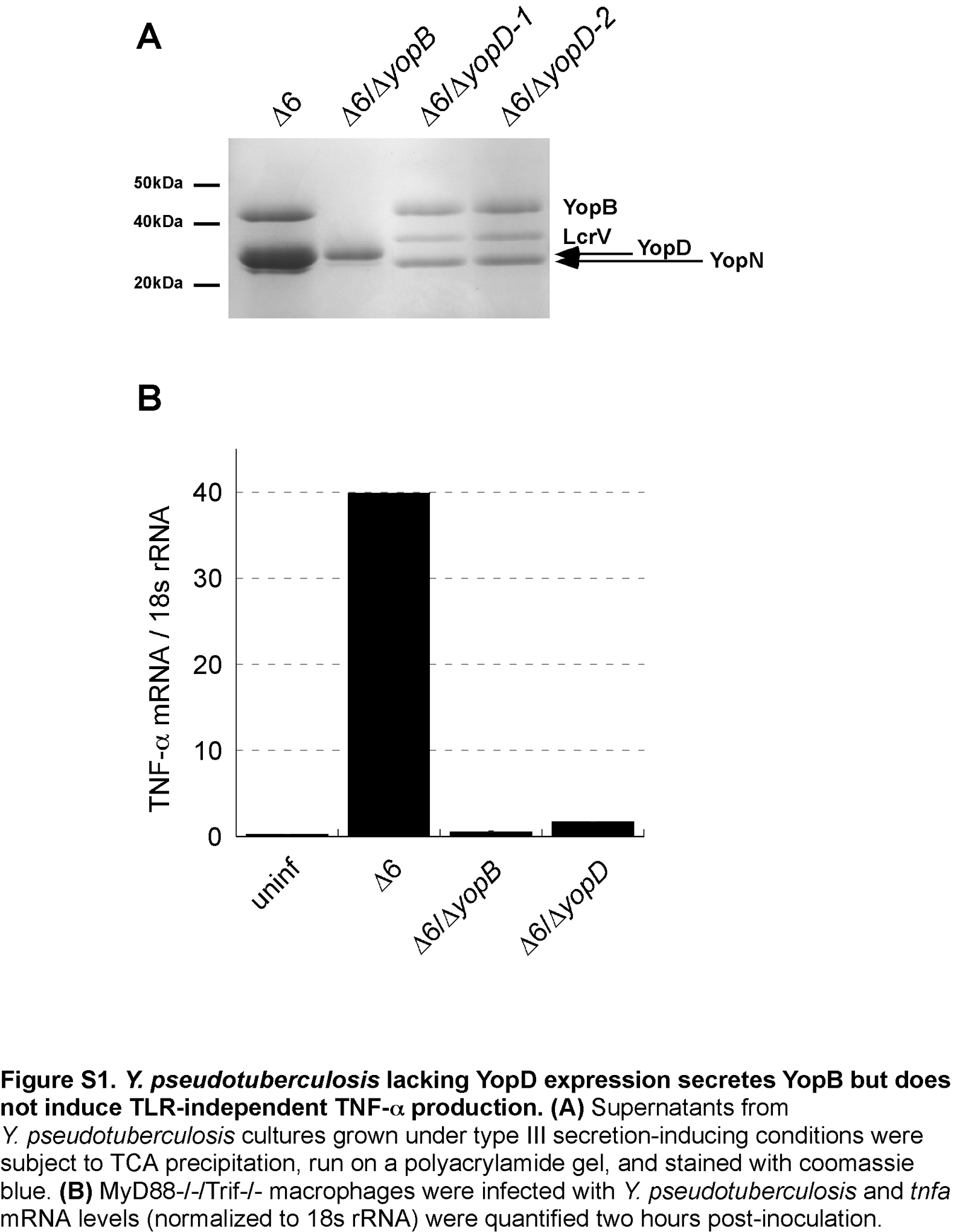

Supplement: Figure S1 — Y. pseudotuberculosis lacking YopD expression secretes YopB but does not induce TLR-independent TNF-α production. (A) Supernatants from Y. pseudotuberculosis cultures grown under type III secretion-inducing conditions were subject to TCA precipitation, run on a polyacrylamide gel, and stained with coomassie blue. (B) MyD88−/−/Trif−/− macrophages were infected with Y. pseudotuberculosis and tnfa mRNA levels (normalized to 18s rRNA) were quantified two hours post-inoculation. (9.69 MB TIF) [file ppat.1000686.s002.tif]
