# Supplementary material for: Innate Immune Recognition of Yersinia pseudotuberculosis Type III Secretion
Source: PLoS Pathog. 2009 Dec 4;5(12):e1000686. doi: 10.1371/journal.ppat.1000686 (PMC2779593; doi:10.1371/journal.ppat.1000686)
Supplement: Figure S2 — Y. pseudotuberculosis expressing a functional T3SS translocator regulates a greater number of macrophage genes than translocator-negative Y. pseudotuberculosis. MyD88−/−/Trif−/− macrophages were infected with Y. pseudotuberculosis Δyop6 or Δyop6/ΔyopB or were left uninfected. Total RNA was isolated 45 minutes, two hours, or four hours post-inoculation and Affymetrix GeneChip Mouse Genome 430 2.0 arrays were used to probe the relative gene expression profiles for each experimental condition. Probe sets regulated by either Y. pseudotuberculosis Δyop6 (diamonds) or Y. pseudotuberculosis Δyop6/ΔyopB (x) at least two-fold over the uninfected condition (p<0.1) were selected. Shown are the log10 of the fold-change values of these selected probe sets. Positive values indicate up-regulation and negative values indicate down-regulation compared to the uninfected condition. For the 45 minute time point, N = 280 (Δyop6) and N = 263 (Δyop6/ΔyopB). For the two hour time point, N = 1248 (Δyop6) and N = 232 (Δyop6/ΔyopB). For the four hour time point, N = 2215 (Δyop6) and N = 338 (Δyop6/ΔyopB). (0.17 MB PDF) [file ppat.1000686.s003.pdf]

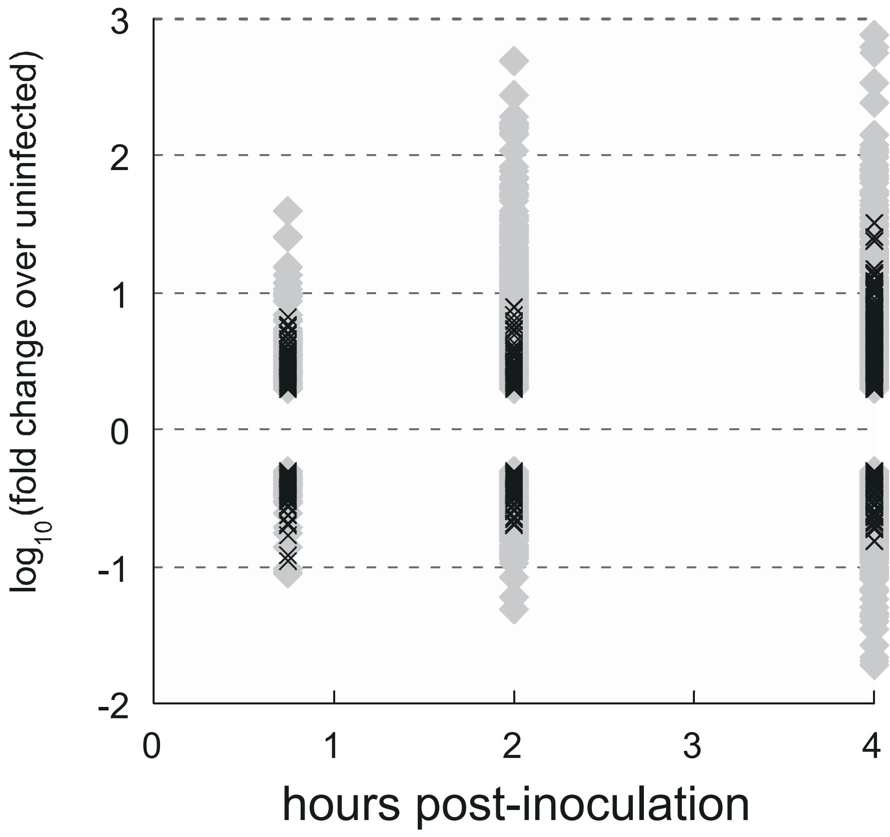

Figure S2. *Y. pseudotuberculosis* expressing a functional T3SS translocator regulates a greater number of macrophage genes than translocator-negative *Y. pseudotuberculosis*. MyD88-/-/Trif-/- macrophages were infected with *Y. pseudotuberculosis* ∆yop6 or ∆6/∆*yopB* or were left uninfected. Total RNA was isolated 45 minutes, two hours, or four hours post-inoculation and Affymetrix GeneChip Mouse Genome 430 2.0 arrays were used to probe the relative gene expression profiles for each experimental condition. Probe sets regulated by either *Y. pseudotuberculosis* ∆yop6 (diamonds) or *Y. pseudotuberculosis* ∆6/∆*yopB* (x) at least two-fold over the uninfected condition ($p<0.1$) were selected. Shown are the log10 of the fold-change values of these selected probe sets. Positive values indicate up-regulation and negative values indicate down-regulation compared to the uninfected condition. For the 45 minute time point, N=280 (∆yop6) and N=263 (∆6/∆*yopB*). For the two hour time point, N=1248 (∆yop6) and N=232 (∆6/∆*yopB*). For the four hour time point, N=2215 (∆yop6) and N=338 (∆6/∆*yopB*).
