# Supplementary material for: Innate Immune Recognition of Yersinia pseudotuberculosis Type III Secretion
Source: PLoS Pathog. 2009 Dec 4;5(12):e1000686. doi: 10.1371/journal.ppat.1000686 (PMC2779593; doi:10.1371/journal.ppat.1000686)
Supplement: Figure S3 — Tnfa mRNA levels peak two hours after inoculation with translocator-positive Y. pseudotuberculosis. MyD88−/−/Trif−/− macrophages were infected with Y. pseudotuberculosis Δyop6 (diamonds) or Δyop6/ΔyopB (circles) and total RNA isolated at 2 hours, 4 hours, or 6 hours post-inoculation. Average tnfa mRNA levels (normalized to 18s rRNA) are shown. (0.10 MB PDF) [file ppat.1000686.s004.pdf]

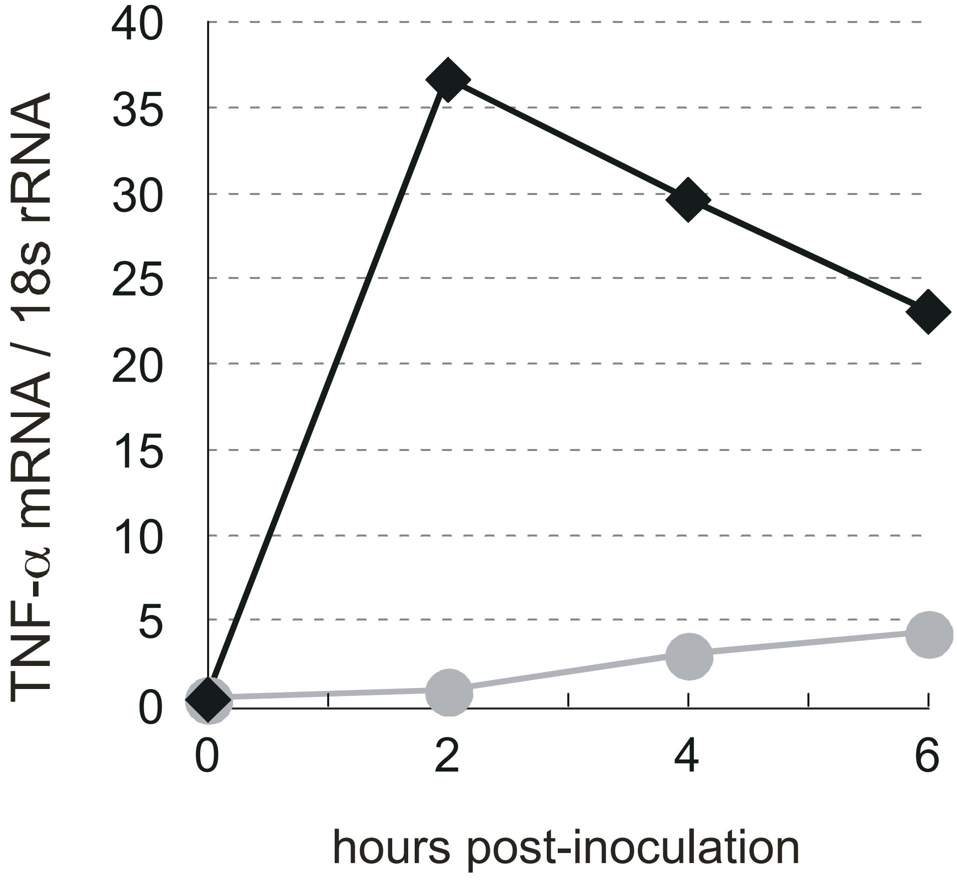

Figure S3. *Tnfa* mRNA levels peak two hours after inoculation with T3SS translocator-positive *Y. pseudotuberculosis*. MyD88-/-/Trif-/- macrophages were infected with *Y. pseudotuberculosis* Δyop6 (diamonds) or Δ6/Δ*yopB* (circles) and total RNA isolated at 2 hours, 4 hours, or 6 hours post-inoculation. Average *tnfa* mRNA levels (normalized to 18s rRNA) are shown.
