# Supplementary material for: Innate Immune Recognition of Yersinia pseudotuberculosis Type III Secretion
Source: PLoS Pathog. 2009 Dec 4;5(12):e1000686. doi: 10.1371/journal.ppat.1000686 (PMC2779593; doi:10.1371/journal.ppat.1000686)
Supplement: Figure S4 — Ifnb mRNA and type I IFN-regulated gene expression are preferentially induced by T3SS translocator-positive Y. pseudotuberculosis. MyD88−/−/Trif−/− macrophages were infected with Y. pseudotuberculosis Δyop6 (diamonds) or Δyop6/ΔyopB (circles) and total RNA isolated at 2 hours (A and B), 4 hours, or 6 hours (A) post-inoculation and qPCR analysis performed. Average ip10 (A) and ifnb (B) mRNA levels (normalized to 18s rRNA) are shown ± sem. (0.11 MB PDF) [file ppat.1000686.s005.pdf]

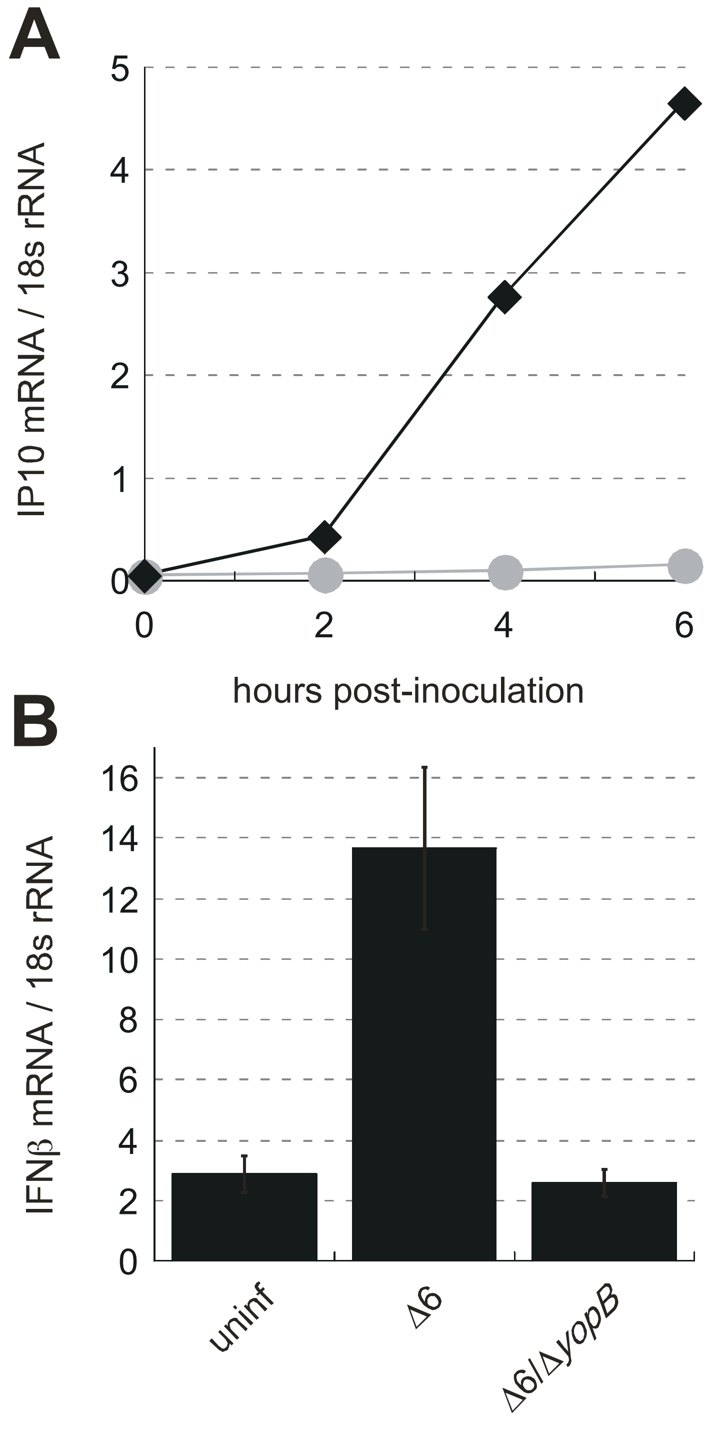

Figure S4. *Ifnb* mRNA and type I IFN-regulated gene expression are preferentially induced by T3SS translocator-positive *Y. pseudotuberculosis*. MyD88-/-/Trif-/- macrophages were infected with *Y. pseudotuberculosis* Δyop6 (diamonds) or Δ6/Δ*yopB* (circles) and total RNA isolated at 2 hours **(A and B)**, 4 hours, or 6 hours **(A)** post-inoculation and qPCR analysis performed. Average *ip10* **(A)** and *ifnb* **(B)** mRNA levels (normalized to 18s rRNA) are shown ± sem.
