# Supplementary material for: Innate Immune Recognition of Yersinia pseudotuberculosis Type III Secretion
Source: PLoS Pathog. 2009 Dec 4;5(12):e1000686. doi: 10.1371/journal.ppat.1000686 (PMC2779593; doi:10.1371/journal.ppat.1000686)
Supplement: Figure S5 — Transfection of type III secreted molecules into primary macrophages does not induce TNF-α or IFNβ. (A,D) MyD88−/−/Trif−/− macrophages were infected with varying amounts of live Y. pseudotuberculosis for two hours. Filtrates from cultures of Y. pseudotuberculosis grown under type III secretion-inducing conditions (B,E) or HKYP (C,F) were transfected into MyD88−/−/Trif−/− macrophages and incubated for two hours. Total RNA was isolated and tnfa (A–C) and ifnb (D–F) mRNA levels (normalized to 18s rRNA) were quantified. Data shown is the average ± sem from one independent, representative experiment and each experiment was repeated for a total of two replicates. * Statistically significant increase (p<0.0001) in cytokine mRNA levels according to the student t-Test compared to uninfected (A) or lipofectamine only (C,F) controls. ** p = 0.0001. *** p<0.0005. Y. pseudotuberculosis strains lacking the regulatory protein YopN hypersecrete Yops [86] and the Δ6/ΔyopN strain was included to test whether T3SS cargo from a hypersecreting Y. pseudotuberculosis strain could trigger IL-8 production. (0.15 MB PDF) [file ppat.1000686.s006.pdf]

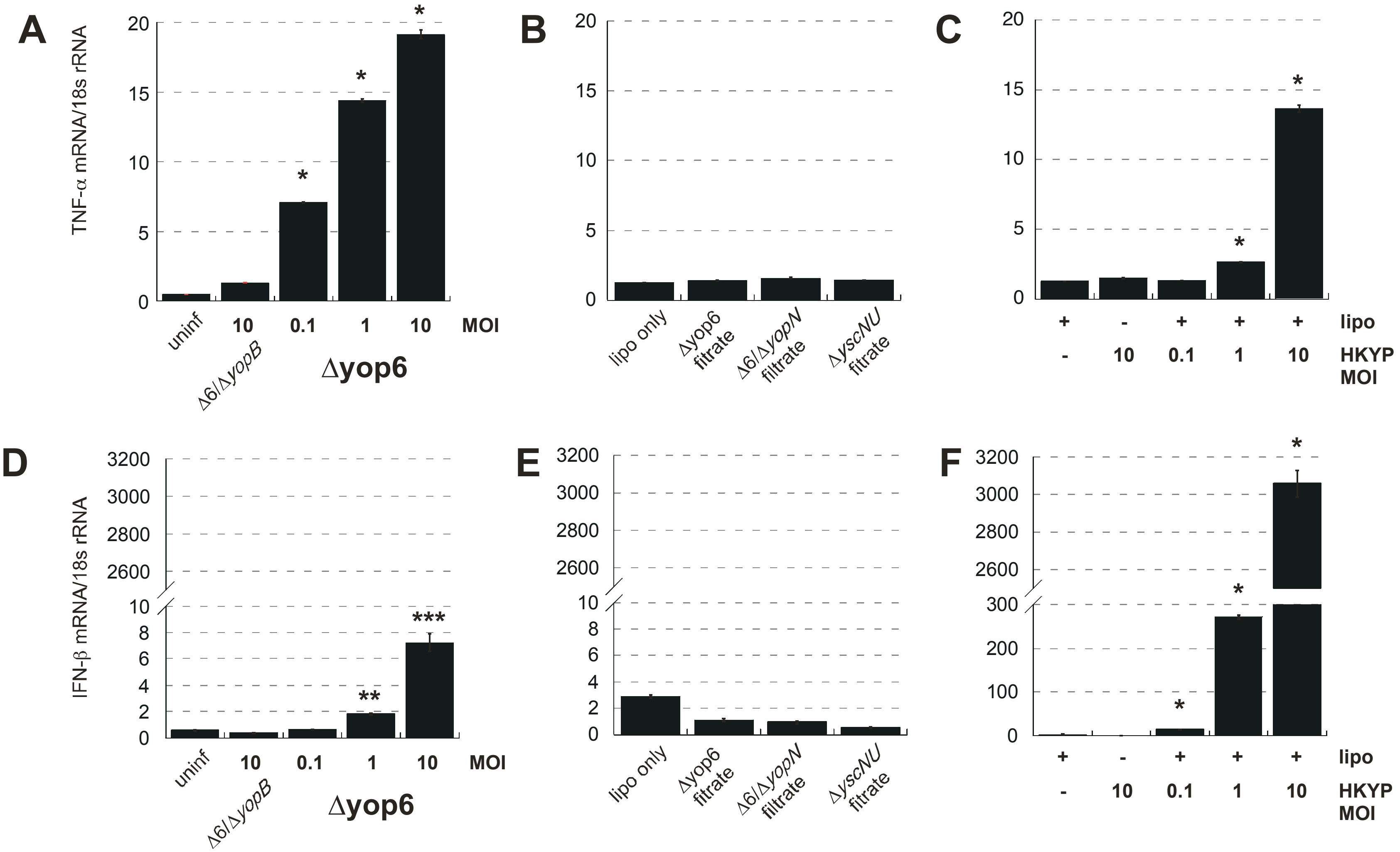

Fig. S5. Transfection of type III secreted molecules into primary macrophages does not induce TNF-α or IFNβ. **(A,D)** MyD88$^{-/-}$/Trif$^{-/-}$ macrophages were infected with varying amounts of live *Y. pseudotuberculosis* for two hours. Filtrates from cultures of *Y. pseudotuberculosis* grown under type III secretion-inducing conditions **(B,E)** or HKYP **(C,F)** were transfected into MyD88$^{-/-}$/Trif$^{-/-}$ macrophages and incubated for two hours. Total RNA was isolated and *tnfa* **(A-C)** and *ifnb* **(D-F)** mRNA levels (normalized to 18s rRNA) were quantified. Data shown is the average ± sem from one independent, representative experiment and each experiment was repeated for a total of two replicates. * Statistically significant increase ($p<0.0001$) in cytokine mRNA levels according to the student t-Test compared to uninfected **(A)** or lipofectamine only **(C,F)** controls. ** $p=0.0001$. *** $p<0.0005$. *Y. pseudotuberculosis* strains lacking the regulatory protein YopN hypersecrete Yops [86] and the Δ6/Δ*yopN* strain was included to test whether T3SS cargo from a hypersecreting *Y. pseudotuberculosis* strain could trigger IL-8 production.
