# Supplementary material for: Innate Immune Recognition of Yersinia pseudotuberculosis Type III Secretion
Source: PLoS Pathog. 2009 Dec 4;5(12):e1000686. doi: 10.1371/journal.ppat.1000686 (PMC2779593; doi:10.1371/journal.ppat.1000686)
Supplement: Figure S6 — Exogenously-added, synthetic RNA synergizes with yopB-expressing, but not yopB-deficient, Y. pseudotuberculosis to induce IFNβ mRNA expression. MyD88−/−/Trif−/− macrophages were infected with Y. pseudotuberculosis Δyop6, Δyop6/ΔyopB, or ΔyopJ in the presence or absence of 1µg/ml poly(I:C). Total RNA was isolated at 2 hours post-inoculation and qPCR analysis performed. Data shown is the average ifnb mRNA level (normalized to 18s rRNA)±sem from one independent, representative experiment and the experiment was repeated for a total of three replicates. (0.10 MB PDF) [file ppat.1000686.s007.pdf]

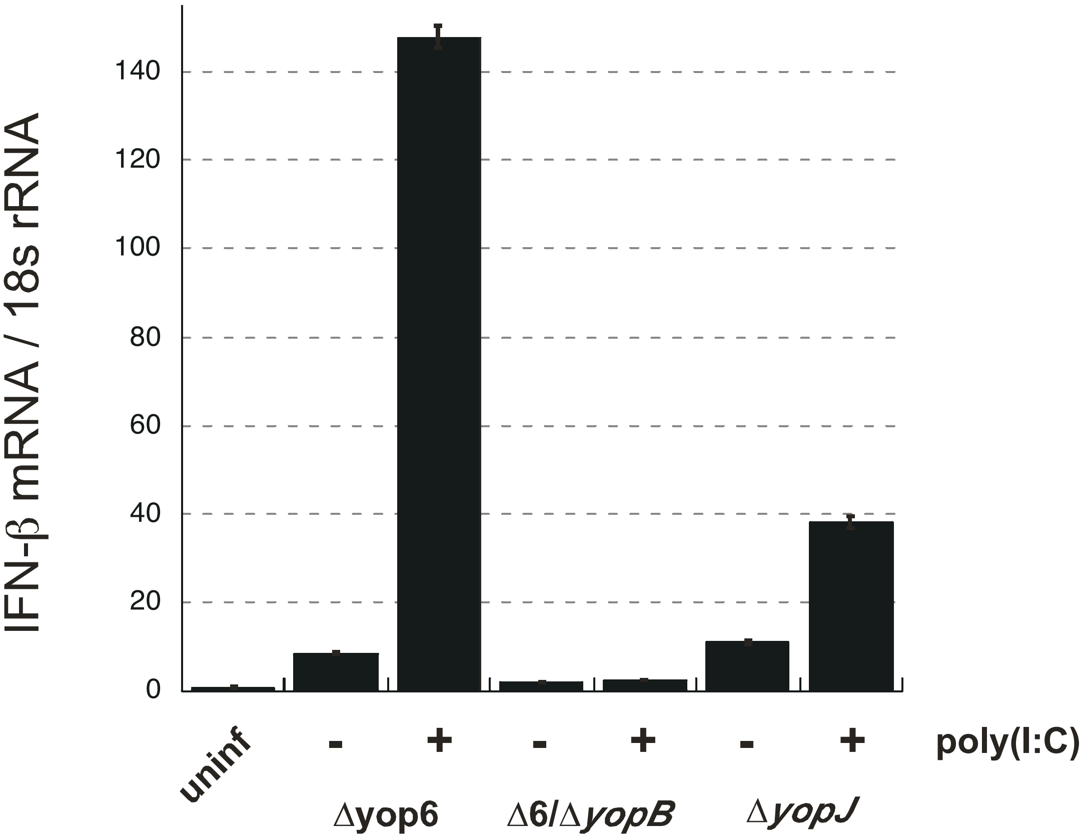

Figure S6. Exogenously-added, synthetic RNA synergizes with *yopB*-expressing, but not *yopB*-deficient, *Y. pseudotuberculosis* to induce IFNβ mRNA expression. MyD88-/-/Trif-/- macrophages were infected with *Y. pseudotuberculosis* Δyop6, Δ6/Δ*yopB*, or Δ*yopJ* in the presence or absence of 1μg/ml poly(I:C). Total RNA was isolated at 2 hours post-inoculation and qPCR analysis performed. Data shown is the average *ifnb* mRNA level (normalized to 18s rRNA) ± sem from one independent, representative experiment and the experiment was repeated for a total of three replicates.
